# Ecological Risk Assessment Is a Living Science: A Study on Heavy Metal(loid) Contamination in Typical Greenhouse Production Systems in Central China

**DOI:** 10.3390/toxics13040312

**Published:** 2025-04-17

**Authors:** Tingting Ma, Peng Wu, Yongchuan Guo, Tian Lei, Shengbo Guo, Huajin Chang, Yongming Luo

**Affiliations:** 1 College of Resource Environment and Tourism, Hubei University of Arts and Science, Xiangyang 441053, China; ttmaxiaotu@126.com; 2 Key Laboratory of Soil and Sustainable Agriculture, Chinese Academy of Sciences, Nanjing 211135, China; ymluo@yic.ac.cn; 3 Jiangsu Rainfine Environmental Science and Technology Co., Ltd., Nanjing 210009, China; woopon@163.com; 4 School of Civil Engineering and Architecture, Hubei University of Arts and Science, Xiangyang 441053, China; 10152@hbuas.edu.cn (Y.G.); 13907278616@163.com (S.G.); 5 Eighth Geological Brigade of Hubei, Xiangyang Geological Environment Monitoring and Protection Station of Hubei, Xiangyang 441000, China; leitian1989@163.com

**Keywords:** facility greenhouse, dietary intake, carcinogenic risk, soil contamination, food safety

## Abstract

To clarify the heavy metal(loid) contamination characteristics and health risk in nine typical greenhouse production areas in Jingmen, Central China, the total concentrations of As, Cd, Cr, Cu, Hg, Ni, Pb, and Zn in 176 soils and 332 vegetables were analyzed. Cadmium (100%) and Ni (4/44) exceeded the Chinese standard limits, while As (36/44), Cd (36/44), Cr (9/44), and Ni (1/44) exceeded the international soil quality standards. The As, Cd, Cr, Ni, and Pb contents in all vegetables were over both the domestic and international standard limitations. The soil pollution levels of Hg and Cd and the potential ecological risk in Zhanghe and Lishi require more attention. Significant levels of Cu, Pb, and Cr in the soil and Hg, Ni, and Cu in edible vegetable parts were suggested to be mainly caused by daily agricultural production management. Although non-carcinogenic and carcinogenic risks to vegetable consumers and greenhouse workers are acceptable across different age groups, more scientific management and remediation must be carried out simultaneously for sustainable production in the future, especially in GD and ZH. Updated standard values for the environment and food, together with the 2023 updated soil geochemical background values, should be applied in time.

## 1. Introduction

As a solution to the food crisis and to meet the increased demand for vegetables, greenhouse facility production has expanded rapidly worldwide, especially in developing countries such as China [1,2]. However, excessive amounts of organic fertilizers, inorganic fertilizers, plastic mulching, and pesticides are used in greenhouses to achieve high vegetable yields, causing soil contamination problems such as heavy metal(loid) pollution [3,4]. The increased and frequent exposure of people to heavy metals is becoming a concern considering their persistence and bioaccumulation in the environment [5,6]. Plants grown in polluted environments can accumulate high heavy metal(loid) concentrations in edible parts such as the roots, leaves, and fruits, which may endanger ecological security and result in human health risks through the food chain [7,8]. In China, the concentrations of As, Cd, Cr, Cu, Hg, Ni, Pb, and Zn in soils are strictly controlled by laws and regulations including the Environmental Quality Evaluation Standards for Farmland of Greenhouse Vegetables Production of China (HJ/T 333-2006) [9], Soil Environmental Quality—Risk Control Standard for Soil Contamination of Agricultural Land of China (GB 15618-2018), and Green Food—Environmental Quality for Production Area of China (NY/T 391-2021) [10]. Heavy metals such as Cd, Cr, Cu, Ni, Hg, Pb, and Zn, as well as metalloids such as As, are recognized as being among the most dangerous contaminants in terms of soil quality and food safety [11]. They can directly damage the cardiovascular system [12], upper gastrointestinal tract [13], and peripheral nervous system [14], and they can induce visceral lesions [15,16] and reproductive system tumors [17]. As, Cd, Cr, Hg, and Pb are classified as certain or probable carcinogens by the International Agency for Research on Cancer [18], and dietary intake is the primary route of human exposure route to heavy metal(loid)s [19].

Soil pollution related to heavy metals and metalloids has been a global problem since the last century; however, the solutions are not as intuitive as those for air or water pollution [20,21]. Heavy metal(loid)s in the soil can be readily transferred to the human body via dermal contact, absorption, inhalation, and ingestion; therefore, the assessment of their potential hazards is essential [22]. As the key development area for the vegetable industry in inland China, Hubei Province occupies 1.94% of the total land area of the country, but feeds 4.30% of the population [23]. Nurtured by the cultivation of the “Jingchu” civilization, Chinese farming culture originated in the middle reaches of the Yangtze River, where prehistoric rice was first discovered. Jingmen City in Central China has become the national modern agriculture demonstration zone, known as “China’s agricultural valley” [24]. In 2017, the Jingmen City government launched a 200 km^2^ ecological vegetable base construction plan [25]. The agricultural areas and vegetable yields in Jingmen rank first in Hubei Province, and the production of large volumes of vegetables and vegetable processing products has made the region an important source of vegetables, not only for people in Wuhan and other cities in Hubei Province, but also for people in other provinces such as Hong Kong [26]. Therefore, there is an urgent need to assess the contamination of target pollutants in both the soils and the vegetables produced to evaluate the health risks to humans by vegetable consumption and other pathways, improve the development and management of the largest representative greenhouse bases of Jingmen City, and ensure vegetable quality and the sustainable use of greenhouse soils.

The aim of this study was to analyze the concentrations of eight target heavy metal(loid)s, namely, As, Cd, Cr, Cu, Hg, Ni, Pb, and Zn, in soil samples and vegetable samples from 186 greenhouses in the nine representative greenhouse vegetable production bases of Jingmen City. The accumulation factors (AFs), geo-accumulation index (Igeo), and PI were calculated to compare the contamination levels of different target elements. By analyzing principal component analysis (PCA) and correlation coefficients between different elements and estimating the carcinogenic risk (CR) and non-CR, as suggested by the U.S. Environmental Protection Agency (USEPA), we discuss the contamination source and potential health risk of heavy metal(loid)s. The results of this study may be used to further control the quality of greenhouse soil, ensure the quality and safety of vegetable production, and mitigate the health risks associated with heavy metal(loid)s originating from greenhouse vegetable production in Central China.

## 2. Materials and Methods

### 2.1. Study Area

Located in the middle and lower reaches of the Hanjiang River, which is the largest branch of the Yangtze River, Jingmen has a total area of 12,400 km^2^ and a population of 3 million, and it includes the largest area of agricultural greenhouses in Hubei Province. The administrative areas of Jingmen City include Jingshan City, Zhongxiang City, Shayang County, Dongbao District, Duodao District, Zhanghe New District, and Qvjialing District. Adhering to the excellent agricultural history of Shennong’s ancestors and experiencing a superior ecological climate suitable for agriculture, Jingmen has become known as the national modern agricultural demonstration zone named “China Agricultural Valley” since 2009, and it is the most representative greenhouse vegetable production base in Hubei Province [27].

**Figure 1:**
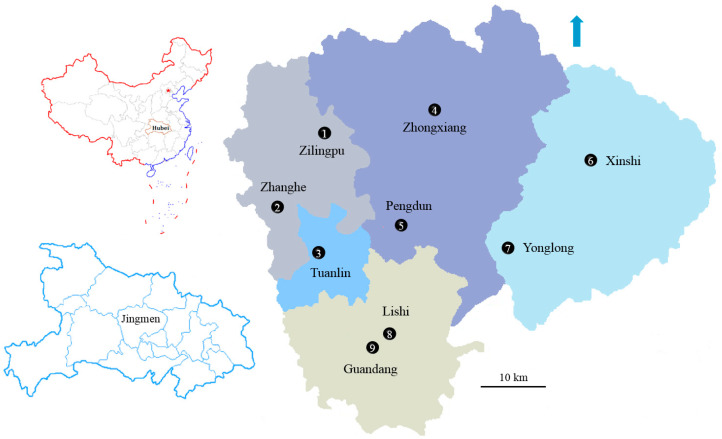
Map of the sampling area in Jingmen City, Hubei Province, China.

Based on the 14th Five-Year Plan for the Agricultural Development of Jingmen City (2021–2025) and the Development Plan of China Agricultural Valley until 2025, nine study areas were screened as distinctive large-scale greenhouse vegetable production areas as follows: (1) the Luoyuan Vegetable Professional Cooperative, a family farm; (2) Shuangfu Ecological Agriculture Development Co., Ltd. (Jingmen, China); (3) Shuangbei Modern Circular Agriculture Demonstration Site; (4) Jinghe Vegetable Planting Specialty Cooperative, a New Rural Construction Industry Support Demonstration Site; (5) Pengdun Wisdom Farm (detailed geographical and production information on areas (1) to (5) are provided in the studies by Ma et al. [26,28]); (6) Jingshan Wujiawan Fruit and Vegetable Picking Garden Planting Farm, which contains fruit picking gardens for tourists, greenhouse vegetable production bases, and agricultural product processing; (7) Jingshan Tianming Family Farm, which is mainly engaged in greenhouse vegetable, fruit, mushroom, and ornamental flower cultivation and sales; (8) Guangxin Vegetable Cooperative Planting Base, a vegetable base with pesticide testing services; and (9) Zhengzhong Modern Rural Ecological Agriculture Farmers Specialized Cooperatives, where the vegetable production process strictly follows the requirements for organic food production. The locations of the nine study areas are shown in Figure 1 and Table S1.

### 2.2. Soil and Vegetable Sampling

A total of 176 soil samples and 332 vegetable samples were collected from 176 out of approximately 1000 greenhouses in the nine study areas of Zilingpu, Zhanghe, Tuanlin, Zhongxiang, Pengdun, Xinshi, Yonglong, Lishi, and Guandang from December 2023 to March 2024, thereby providing a total of 508 samples (Figure 1 and Table S1). Each soil sample was collected from the top layer (0–15 cm) with a soil corer, and the mean value of five randomly selected samples from the same greenhouse in a quincunx pattern in the non-marginal region was calculated. Vegetable samples were collected from the same position as the soil samples by cutting down randomly screened leaves and fruits from non-adjacent and non-marginal plants in quintuplicate from each greenhouse. All soil and vegetable samples were analyzed in triplicate (a total of 1524) in a laboratory. The soil samples were air-dried at 25 °C and ground to pass through a 2 mm sieve after being stored in linen sampling bags and returned to the laboratory. The vegetables were thoroughly washed and rinsed with deionized water, wiped dry with tissue paper, oven-dried at 105 °C for 30 min to destroy enzymes, and then oven-dried to constant weight at 85 °C to minimize the volatilization of organic constituents [29]. The plant samples were then homogenized using a stainless-steel mill before determining the concentrations of the target pollutants. All collected soils were alfisols. The basic physico-chemical properties, including soil pH and organic matter content, were determined [30] and are listed in Table S1 along with an overview of the study areas.

### 2.3. Soil and Plant Sample Analysis

The soil and plant samples were digested in a Multiwave GO microwave digestion system (Anton Paar GmbH, Graz, Austria) and then analyzed using inductively coupled plasma mass spectrometry (Thermo X7, Thermo Scientific, Waltham, MA, USA). The certified reference materials GBW07404 (GSS-4), GBW07387 (GSS-31), and GBW07603 (GSV-2), provided by the Institute of Geophysical and Geochemical Exploration (Langfang, Hebei Province, China), were used as part of the quality control process in the soil and plant analysis, respectively. The recovery results for the standard reference materials were 88.65–109.92%, 90.97–101.58%, and 93.41–107.87%, respectively, all within the published confidence intervals.

The total Cd, Cr, Cu, Ni, Pb, and Zn contents in soil samples were determined after digestion, in accordance with Ma et al. [12]. The digestion procedure for As and Hg was modified and accomplished as follows [31]. Aliquots of 0.5000 ± 0.0005 g soil samples were transferred directly into separate microwave polytetrafluoroethylene (PTFE) vessels and mixed with 4 mL of HNO_3_, 2 mL of HCl, and 1 mL of HF before being sealed and placed in a microwave oven. The digestion power was increased to 300 W in 5 min (held for 5 min), increased from 300 W to 600 W in 5 min (held for 10 min), and finally cooled (for 15 min). The most critical step in the digestion of As and Hg compared with that of the other six heavy metals was to gently remove the acid in the digestion system by heating the digestion solution to avoid drying and the subsequent loss of As and Hg. The digests were filtered into 50 mL volumetric flasks, brought to volume using Milli-Q ultrapure water (18.2 MΩ·cm), transferred to plastic bottles, and stored at 4 °C before analysis.

For the vegetable samples, both edible parts and leaf samples were analyzed, respectively. The total As, Cd, Cr, Cu, Hg, Ni, Pb, and Zn contents were determined following the same digestion procedures conducted by Nardi et al. [32] with modification, as follows. Aliquots of 0.2500 ± 0.0005 g vegetable powder were mixed with 3 mL of 60% HNO_3_ and 2 mL of HF before digestion in 50 mL PTFE vessels in a microwave oven at medium pressure and 200 °C for 12 h. A colorless solution was obtained, brought to 50 mL using ultrapure water, and stored at 4 °C before analysis.

### 2.4. Data Analysis

#### 2.4.1. Geo-Accumulation Index

Following the method of Müller [33], the index of geo-accumulation (Igeo), which measures the contamination of elements in sediments, was calculated as follows:

Igeo = log2(C_soil_/k C_b,i_) (1)

where C_soil_ is the determined concentration of the element i in a sample, C_b,i_ is the natural background concentration of the element i, and k is the correction factor for the possible variations in the background values from lithogenic effects. The geochemical backgrounds of the eight target elements are listed in Table 1 [34,35]. The classification of the degree of pollution based on the Igeo values is shown in Table S2.

#### 2.4.2. Accumulation Factor

As one of the key components contributing to human exposure to heavy metal(loid)s is via the food chain, the AF or biological absorption coefficient, which provides information about the contamination pathways of plants from polluted soil, were calculated for different vegetable types in the edible part and in soils based on dry weight from the nine study areas [36] as follows:

AF = C_vegetable_/C_soil_ (2)

where C_vegetable_ and C_soil_ represent the total concentration of target element *i* in the edible part of vegetables and in soils based on dry weight.

#### 2.4.3. Potential Ecological Risk Index

As the single pollution index, PI was calculated to evaluate the degree of contamination of each target element [34], as follows:

PI = C_soil_/C_b,i_ (3)

where C_soil_ is the concentration of the target element in the soil, and C_b,i_ is the corresponding background values of the elements in the soil of the study area shown in Table 1.

The Nemerow integrated pollution index (NIPI) was used to assess the overall pollution status of all target elements in the soils from the study areas [37], and it was calculated as follows:

(4) $$NIPI=\sqrt{[{\left(P_{\max}\right)}^{2}+{\left(P_{mean}\right)}^{2}]/2}$$

where P_max_ is the maximum value of the PI, and P_mean_ is the average value of the PI.

The potential ecological risk index (RI) proposed by Håkanson [38] was used to evaluate the ecological risk posed by the target elements in the soils from the study areas, and it was calculated using the following formula:

(5) $$RI=\sum_{i=1}^{n}E_{r}=\sum_{i=1}^{n}\left(T_{r}\times PI\right)$$

where E_r_ is the ecological risk of the target element and T_r_ is the toxicity response coefficient of the target element. For the eight target elements As, Cd, Cr, Cu, Hg, Ni, Pb, and Zn, the toxicity response coefficients were 10, 30, 2, 5, 40, 5, 5, and 1, respectively [38]. The classification of the degree of pollution based on the PI, NIPI, E_r_, and RI values are listed in Table S2 [39].

#### 2.4.4. Non-Carcinogenic Risk Assessment

Potentially toxic heavy metal(loid)s in soils can directly affect human health; therefore, hazard quotient (HQ) values are usually employed to evaluate the potential health risks stemming from dietary intake, soil ingestion, inhalation, or dermal contact with toxic elements. However, the potential non-CR and CR of toxic elements are different for adults and children based on their behavior and physiology. In this study, participants were divided into the following six groups: (1) young children (0–6 years old), (2) young adolescents (7–14 years old), (3) adolescents (15–19 years old), (4) male adults (19–59 years old), (5) female adults (19–59 years old), and (6) older people (60–79 years old). This was to show the age-dependent variations in non-carcinogenic risks [40,41]. According to the methodology provided by the USEPA for non-CR assessment [42], the equation for the HQ calculation of a single target pollutant is given by Ma et al. [26]. Here, different exposure routines were considered in the calculation of non-carcinogenic risks and carcinogenic risks, including vegetable intake, soil oral digestion, soil dermal contact, and soil inhalation. Vegetable intake health risks were individually evaluated as the model of vegetable consumers, while soil ingestion, dermal contact, and inhalation exposure routes were evaluated together as the model of greenhouse workers.

HQ values can be calculated according to the following formulas:

HQ_intake_ = C_vegetable_ × IR × EF_vegetable_ × ED_vegetable_ × CF/BW/AT/RfD (6)

HQ_ingestion_ = C_soil_ × IR_soil-ingestion_ × EF_soil_ × ED_soil_ × CF/BW/AT/RfD (7)

HQ_dermal_ = C_soil_ × SA × ADF × ABS × EF_soil_ × ED_soil_ × CF/BW/AT/RfD (8)

HQ_inhalation_ = C_soil_ × ET × EF_soil_ × ED_soil_/PEF/AT/RfC/24 (9)

According to the differences in vegetable consumption and vegetable types, the IR values for daily vegetable intake for children and adults were identified, and the duration of exposure for the different age groups was set to 4, 12, 17, 40, and 70 years, respectively. Considering the frequency of greenhouse workers bringing children to work with them, the exposure frequency was set as one third that of adults. The other parameters were generally the same as the original documents. All of the values are listed in Table S3.

To evaluate the overall potential non-CR from all target pollutants, the chronic effects of multiple elements were assessed using the hazard index (HI), provided by the Guidelines for Health Risk Assessment of Chemical Mixtures of USEPA [43], as follows:

HI = HQ1 + HQ2 + … + HQn(10)

The guidelines for interpreting the HQ are as follows: HQ < 1, hazard is low; 1 < HQ < 10, hazard is moderate; and HQ > 10, hazard is high [44]. The guidelines for interpreting the HI are as follows: HI < 1, there are no non-carcinogenic effects on the target population; and HI > 1, non-carcinogenic effects are considered [44].

#### 2.4.5. Carcinogenic Risk Assessment

The CR, as the cancer probability in a population or the number of individuals with cancer in a certain population, was calculated as follows [45]:

CR_intake_ = C_vegetable_ × IR × EF_vegetable_ × ED_vegetable_ × CF/BW/AT_lifetime_ × SFO (11)

CR_ingestion_ = C_soil_ × IR_soil-ingestion_ × EF_soil_ × ED_soil_ × 10^−6^/BW/AT_lifetime_ × SFO (12)

CR_dermal_ = C_soil_ × SA × ADF × ABS × EF_soil_ × ED_soil_ × 10^−3^/BW/AT_lifetime_ × SFO (13)

CR_inhalation_ = C_soil_ × ET × EF_soil_ × ED_soil_/PEF/AT_lifetime_/24 × IUR × 10^3^ (14)

The oral slope factor of carcinogens (mg/kg)/d was obtained from the USEPA Integrated Risk Information System database [44]. As people live longer, lifetime days were modified from 25,550 days (70 years) to 28,835 days (79 years) in this study. In general, a CR value (unitless) lower than 10^−6^ is considered to be negligible, a value above 10^−4^ is considered unacceptable, and a value between 10^−6^ and 10^−4^ is considered an acceptable range [45]. Details of all of the other parameters are listed in Table S3.

### 2.5. Source Apportionment

Positive matrix factorization (PMF) is the preferred option of the receptor method, which is a mathematical calculation approach used to quantify the source and contribution of samples in recent years. The PMF method was used to calculate the source contribution and profiles of different heavy metal(loid)s according to the methods of Hua et al. [6].

### 2.6. Statistical Analysis

All of the concentrations of the eight target elements were listed as the average values of quintuplicate measurements in each greenhouse ± the standard deviation. All statistical analyses were performed using the SPSS software package (version 20.0; SPSS, Chicago, IL, USA), Microsoft Excel 2016, and the Origin 2024 SR1 software package. A Pearson correlation analysis was applied to reveal the relationships between the concentrations of different target elements in different types of samples with a two-tailed test of significance. In all tests, a significant difference was considered if *p* < 0.05, and an extremely significant difference was considered if *p* < 0.01. PCA was conducted to clarify the principal components and eigenvectors in the different types of samples from the nine study areas. Differences were assessed using one-way analysis of variance followed by Tukey’s test to compare the calculated parameters. Heatmaps of the HI values were plotted to show the non-CR values, and grouped scatter plots were used to show the CR values of target elements to different groups of people using Origin 2024.

## 3. Results and Discussion

### 3.1. Contamination of Target Elements in Soils

The concentrations of the eight target elements, namely, As, Cd, Cr, Cu, Hg, Ni, Pb, and Zn, in the greenhouse soils from the nine study areas in Jingmen City are listed in Table 2. The concentrations of the target heavy metal(loid)s varied widely in the soil samples across the different study areas in the order of Zn > Cr >Ni > Pb > As > Cd > Cu > Hg. The concentration ranges were 10.090–17.690, 1.120–3.420, 10.120–83.200, 0.130–0.220, 0.016–0.097, 25.130–43.020, 14.960–29.620, and 69.190–195.970 mg/kg for As, Cd, Cr, Cu, Hg, Ni, Pb, and Zn, respectively (Table 2). The contamination of different target elements in the soil samples from the nine study areas was not uniform. Compared with the reference guideline values of the target elements, the Cd concentrations in all of the soil samples exceeded the Soil Environmental Quality—Risk Control Standard for Soil Contamination of Agricultural Land of China (GB 15618-2018) [46] (0.3 mg/kg), the Chinese target value for greenhouse soil environments (HJ/T 333-2006) (pH < 7.5, 0.3 mg/kg; 7.5 < pH, 0.4 mg/kg) [9], exceeding the Limits of Green Food Origin Environmental Quality Standards of China (NY/T 391-2021) (pH < 7.5, 0.3 mg/kg; 7.5 < pH, 0.4 mg/kg) [10] by 2.9 to 22.1 times. Compared with the Japanese guidelines (0.4 mg/kg) [47] and the Canadian Soil Quality Guidelines (1.4 mg/kg) [48], the excess rate of Cd concentrations in all soils are 100% and 86.36% (38/44), respectively. Although As concentrations in all soils are lower than the Chinese limitation values or guidelines (20–40 mg/kg), 81.82% (36/44) are higher than the Canadian Soil Quality Guidelines (12 mg/kg) [48] and 20.45% (9/44) exceed the Japanese guidelines (15 mg/kg) [47]. For Cr, 20.45% (9/44) exceed the Canadian Soil Quality Guidelines (64 mg/kg) [48]. For Ni, 4 out of 44 (9.09%) samples exceed the Chinese target value for greenhouse soil environments (HJ/T 333-2006) (40 mg/kg) [9] and 1 out of 44 exceed the Canadian Soil Quality Guidelines (45 mg/kg) [48].

The rapid expansion of Chinese greenhouses has resulted in significant problems with soil quality, including soil acidification, salinization, and pollution from heavy metal(loid)s, pesticides, antibiotics, and nutrients [49]. The problem of Cd pollution is quite prominent among the eight target heavy metal(loid)s according to both the national and international soil environmental quality control standards. Cd is often the most severe pollutant, although the contamination level of Cd in this study (1.120–3.420 mg/kg) is at a medium to high level compared with some other domestic greenhouse soils. It is lower than that in Baiyin Gansu (2.43–5.83 mg/kg) [49] but higher than that in Jingyang, Shaanxi, China (0.12–1.07 mg/kg) [50], the Beichuan River basin (ND-2.33 mg/kg) [6], Shaanxi (0.078–0.54 mg/kg) [51], the Jinyuan District of Taiyuan City (0.07–0.76 mg/kg) [52], and Shenyang (0.09–0.402 mg/kg) [7], among others. In a review of soil contamination in plastic shed vegetable production areas in China, it was found that Cd not only exceeded the maximum limit value (0.06–179.4 mg/kg), but it also had the highest average geo-accumulation index at the extremely polluted level, with an approximate rate of 1.2% [11]. Cd soil contamination gradually began to emerge in Jingmen, and the environmental quality of the soil in greenhouse systems is now a serious concern in China, especially in terms of Cd pollution. The greenhouse vegetable production systems currently in use have led to the accumulation of Cd, Cu, and Zn in organic greenhouse soils in Nanjing, East China [53]. Pb, Cd, and Ni concentrations in greenhouse soils in Xining were found to be below the limit values and were considered safe, but Cd and Pb concentrations in 72.4% and 35.5% of the soil samples in greenhouses in northern China were higher than the Grade II value of the Environmental Quality Standard for Soils (GB15618) [54]. The greenhouse soils had higher concentrations and percentages of As, Cr, Cu, Ni, and Zn in bio-accessible fractions than field soils in Gansu, western China [3]. Long-term vegetable production can lead to significant cumulative effects of Cd, Zn, and Cu in soils, as observed in northeast China [1]. Zinc has also been detected as pollutant, with the highest concentrations in greenhouse fields in Hamedan Province, Iran [55].

For commonly planted vegetables, the comprehensive pollution degrees were also different. The accumulation of all the target elements in the soil of cucumbers (Figure S1a) was in the order of Pengdun > Tuanlin > Guandang > Lishi > Yonglong > Zilingpu (green) > Zilingpu (yellow) > Xinshi. For eggplants, green chilis, and tomatoes, the orders were Guandang > Zhanghe > Pengdun > Tuanlin > Lishi > Yonglong > Zilingpu > Xinshi (Figure S1b); Zhanghe > Guandang > Pengdun > Lishi > Yonglong > Zhongxiang > Zilingpu > Xinshi (Figure S1c); and Tuanlin > Guandang > Pengdun > Lishi > Zilingpu > Yonglong > Xinshi (Figure S1d), respectively.

Based on the former and updated soil background values from 1990 [34] to 2023 [35], the calculation results for the Igeo values of different target contaminants also differed. Based on the early published geochemical backgrounds of the target elements [34], As, Cr, and Pb could be classified as unpolluted (Figure S2a). The Igeo values of Hg (>5), Cd (1~5), Ni (1~3), Cu (1~3), and Zn (−1~1) were higher than zero, indicating their contamination degree was extremely polluted, strongly polluted, moderately to strongly polluted, moderately to strongly polluted, and unpolluted to moderately polluted, respectively (Figure S2a). However, based on the updated geochemical backgrounds of target elements in Hubei Province [35], the concentrations of As, Cr, and Pb in the samples classified them as unpolluted (Figure S2b). The Igeo values of Ni (−1~1) were also at an unpolluted level. Although the Igeo values of Zn (−1~2) (Figure S2b) were also lower than for Zn (−1~1) (Figure S2a), the pollution classification degree did not change. Cd and Hg were the main limiting factors of the soil environmental index, with similar conclusions being drawn for vegetable greenhouses in Jingyang County in Shaanxi Province, China [56]. Comparing the Igeo results based on the different geochemical backgrounds of the target elements shows that the definition of the nickel pollution level has changed significantly, mainly due to the updated soil nickel background value being considerably increased (from 3.73 to 30.7–37.5).

**Figure 2:**
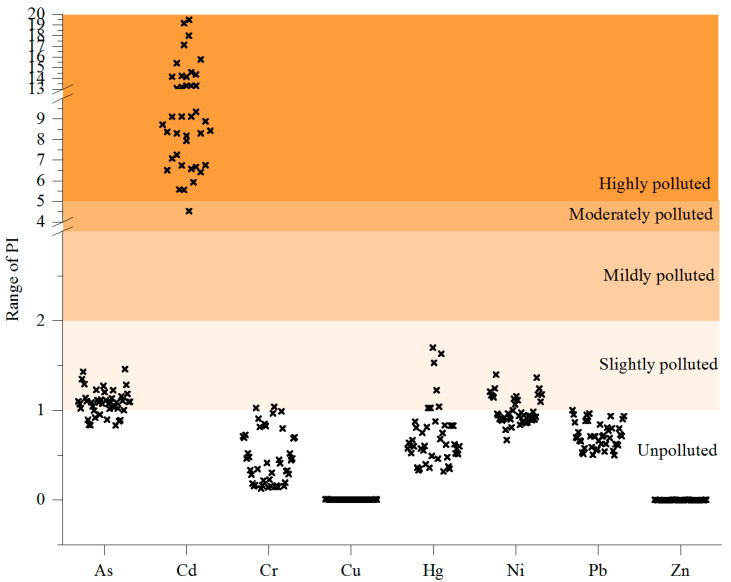
Single pollution index (PI values) of the eight target pollutants in different greenhouse soils (*n* = 176). Black “x” is the individual PI value of each pollutant in all the investigated soils. Different colours indicate the pollution levels are “Unpolluted”, “Slightly polluted”, “Mildly polluted”, “Moderately polluted” and “Highly polluted” when PI values are between 0–1, 1–2, 2–4, 4–5 and over 5.

The PI values follow the order of Cd > As > Ni > Hg > Cr > Pb > Cu > Zn (Figure 2). Based on the PI values, Cd had the highest contamination level, indicating the samples were highly polluted; As, Hg, and Ni presented slight pollution; and Cr, Cu, Pb, and Zn concentrations indicated the samples were generally unpolluted. Based on a previous study conducted in Hubei Province in 2016, the single PI of Cr and Cu in Jingzhou City and Cr in Huanggang City exceeded and was near the warning level, respectively, but in other urban areas of Wuhan, Yichang, Jingmen, Enshi, Shiyan, and Xianning, the levels were safe [57] and were much lower than in our study.

The mean Nemerow integrated pollution index (NIPI) values of the nine investigated areas follow the order of Zhanghe (3.70) > Lishi (3.69) > Guandang (3.40) > Zilingpu (3.39) > Zhongxiang (3.29) > Tuanlin (3.12) > Pengdun (2.83) > Yonglong (2.74) > Xinshi (2.24), based on the mean values calculated using the geochemical backgrounds of the target elements published in 1990 [34] (Table S4). The soils from the greenhouses in the first six study areas are severely polluted, and those from the latter three are moderately polluted. However, based on the mean NIPI values calculated using the geochemical backgrounds of the target elements updated in 2023 [35], the sequence changes to Lishi (2.79) > Guandang (2.61) > Zhanghe (2.59) > Zilingpu (2.17) > Tuanlin (2.06) > Zhongxiang (1.76) > Yonglong (1.58) > Pengdun (1.39) > Xinshi (1.18) (Table S4). Not only are the NIPI values of the different areas lower, but the general contamination levels have also decreased to a moderate level for five out of nine samples and a low level for the remaining four. The values of As, Cr, Cu, 37/44 of Hg (84.09%), Ni, Pb, and Zn in the greenhouses of the nine study areas indicate low risk (Table S5). However, the proportions for the disastrous levels and high levels are 20/44 (45.45%) and 24/44 (54.55%) for Cd and 0% and 7/44 (15.91%) for Hg, based on the calculations for the new geochemical backgrounds of the target elements [35]. The results show that the risk level of 3/44 for Cd (6.82%) has reduced from the disastrous risk to high risk level, and 38/44 for Ni (86.36%) has reduced from a moderate risk level to a low risk level, but the risk level of 4/44 for Hg (9.09%) has increased from a low risk level to a moderate risk level (Table S5). As a scale for calculating pollution levels, the geochemical backgrounds of different elements should be updated as soon as possible after a certain period of time, because the impact of human activities on the background value of the natural environment is more direct and intense. Thirty years is too long a period to wait for the renewal of these data. In addition to regular soil pollution surveys, the environmental management department in China should also update these background data in a timely manner in order to better understand the pollution status and risk levels of different elements in different regions.

In the different study areas (Table S6), the contamination levels based on the range of the RI values are as follows: Zhanghe (429.92–686.36, considerable to high risk) > Lishi (424.58–605.25, considerable to high risk) > Zhongxiang (368.06–581.54, considerable risk) > Zilingpu (350.79–553.58, considerable risk) > Tuanlin (342.91–541.76, considerable risk) > Guandang (455.54–526.50, considerable risk) > Yonglong (376.33–392.95, considerable risk) > Pengdun (286.79–396.84, moderate to considerable risk) > Xinshi (258.22–301.38, moderate to considerable risk). The updated RI values follow the order of Zhanghe (366.41–602.35, considerable to high risk) > Lishi (437.70–635.64, considerable to high risk) > Guandang (366.82–556.60, considerable risk) > Yonglong (302.37–319.20, considerable risk) > Zilingpu (285.20–482.56, moderate to considerable risk) > Tuanlin (290.10–469.79, moderate to considerable risk) > Zhongxiang (239.65–372.97, moderate to considerable risk) > Pengdun (186.33–252.35, moderate risk) > Xinshi (196.49–233.83, moderate risk). The degree of risk for Zilingpu, Tuanlin, Zhongxiang, Pengdun, and Xinshi have been downgraded based on the calculations using the new geochemical backgrounds for the target elements (Table S6). The general moderate to considerable degree of risk of the eight target elements in typical greenhouses in Jingmen is better than that in the greenhouse vegetable production systems in Jingyang, Shaanxi, where the RI values indicated that 63.7 and 14.3% of the area was at a moderate and high ecological risk, respectively [50].

### 3.2. Contamination of Target Elements in Vegetables

The concentrations of the eight target elements in the edible parts of vegetables in greenhouses in Jingmen based on dry weight are listed in Table S7. The average concentrations (mg/kg) of heavy metal(loid)s varied among the different vegetable species and sampling areas in the following order: Zn (35.14) > Pb (7.21) > Cr (6.94) > As (6.33) > Ni (5.70) > Cd (0.83) > Cu (0.048) > Hg (0.0096). The concentration ranges were 3.260–17.570, 0.440–3.040, 3.120–28.030, 0.017–0.103, 0.005–0.018, 1.390–12.710, 4.540–11.520, and 14.130–54.730 mg/kg for As, Cd, Cr, Cu, Hg, Ni, Pb, and Zn, respectively (Table S7). In all nine study areas, cucumber (8), eggplant (8), green chili (8), and tomato (7) are the four most popular vegetables grown in greenhouses. For commonly planted vegetables, the accumulation of all of the target elements in the edible parts of cucumbers (Figure S1e) was in the order of Guandang (mini) > Zilingpu (green) > Lishi > Pengdun (mini) > Yonglong > Tuanlin > Zilingpu (yellow) > Xinshi. For eggplants, green chilis, and tomatoes, the orders were Guandang (purple ball) > Lishi (white) > Zilingpu (green) > Yonglong (long purple) > Tuanlin (long purple) > Xinshi (purple) > Pengdun (purple) > Zhanghe (long purple) (Figure S1f); Guandang > Lishi > Yonglong > Pengdun > Xinshi > Zhongxiang > Zhanghe > Zilingpu (Figure S1g); and Pengdun (cherry) > Lishi > Guandang (cherry) > Tuanlin (cherry) > Yonglong > Xinshi > Zilingpu (Figure S1h), respectively. Except Cu, 100% of As, Cd, Cr, Ni, and Pb and 40.91% of Hg (18/44) in the vegetable edible parts exceeded the standard values of the Chinese Maximum Levels of Contaminants in Foods (GB2762–2022) [58], the World Health Organization (WHO) and Food and Agriculture Organization (FAO) (2011) [59] permission limits, and the regulations regarding the maximum levels of certain contaminants in food recommended by the European Union (EU) (2023) [60] (Table S7). The EU always gives precise recommendations on the limitation values of different contaminants in food. However, as per the EU 2023/915 regulations regarding the maximum levels of certain contaminants in food, recommendations are only made for Cd and Pb in vegetables [60]. Approximately 15.91% of Zn (7/44) in vegetables’ edible parts was below the WHO and FAO [59] permission limits (20 mg/kg). The exceedance multiples for the different elements were approximately 12.66, 4.15, 3.02, 3.8, and 14.42 for As, Cd, Cr, Ni, and Pb, respectively. To deal with the pollution of vegetables caused by soil pollution, the screening of low-accumulation vegetables based on combined pollution from multiple heavy metals in the edible parts has become an important solution [61].

For leaves, the concentrations of heavy metal(loid)s in vegetable species and sampling areas were in the following order: As > Zn > Cr > Pb > Ni > Cd > Cu > Hg. The mean concentration ranges were 250.990–1720.670, 0.460–3.030, 2.160–19.470, 0.018–0.107, 0.013–0.127, 0.460–14.800, 1.960–14.970, and 24.91–224.45 mg/kg for As, Cd, Cr, Cu, Hg, Ni, Pb, and Zn, respectively (Table S8). The average concentration of As in the leaves was nearly 100 times higher than that in the edible parts of the vegetables. It has been reported that tuberous vegetables accumulate more As than leafy vegetables, and leafy vegetables accumulate more As than fruity vegetables [62]. Cd concentration in the edible parts of pak choi peaks during the early slow growth period due to its higher mobility compared to other metal(loid)s [49]. Leafy vegetables have higher concentrations and transfer factors of heavy metals than root and fruit vegetables, especially Cd, as concluded from the investigation of a typical greenhouse vegetable production system in Jiangsu Province [63]. The Cd concentration in 21% of leaf samples and the Pb concentration in 38% of leaf samples were above the thresholds established for yerba mate leaves in South America [64]. However, in this study, this trend was not as clear, except for that of As in all the collected vegetable samples.

As shown in Figure S3, large variations in the AFs based on the total soil heavy metal(loid) concentrations and edible vegetable parts were observed among different vegetable species and elements. The average AFs in all of the vegetables were in the order As > Cd > Zn >Pb > Cr > Cu > Hg > Ni. Considering the total AFs of heavy metal(loid)s in vegetables, the highest AF was observed in Chinese cabbage in Zhongxiang (4.70), whereas the lowest was observed in long purple eggplant in Zhanghe (1.86) (Table S9). The classification of the contamination level by means of AFs is minor between 1 and 3, moderate between 3 and 5, and moderately severe between 5 and 10. Thus, the contamination levels of the vegetables in the nine typical greenhouse production areas are at a minor to moderate level. The sum of average AFs of all target elements in cucumbers were in the order of Zilingpu (yellow) (2.71) ≈ Guandang (mini) (2.71) > Zilingpu (green) (2.68) > Lishi (2.63) > Yonglong (2.48) > Xinshi (2.31) > Tuanlin (2.19) > Pengdun (mini) (2.16). Those for eggplants, green chilis, and tomatoes were in the order of Zilingpu (2.72) > Yonglong (2.52) ≈ Lishi (2.52) > Guandang (2.50) > Xinshi (2.31) > Tuanlin (2.00) > Pengdun (1.97) > Zhanghe (1.86); Zhongxiang (3.26) > Zhanghe (2.62) > Yonglong (2.58) > Lishi (2.54) > Xinshi (2.43) > Guandang (2.38) ≈ Pengdun (2.38) > Zilingpu (2.00); and Pengdun (cherry) (3.75) > Tuanlin (cherry) (3.59) > Lishi (2.76) > Guandang (cherry) (2.57) > Yonglong (2.52) > Xinshi (2.41) > Zilingpu (2.17), respectively. Moreover, 5/44 samples for As, 1/44 for Cd, 3/44 for Cr, and 2/33 for Hg were found to be at a minor contamination level in this study. However, in a study of greenhouse-cultivated soils in Northern Jordan, 23.4% and 38.3% of the sampling sites showed moderate contamination for Pb and Cd, while 8.5% showed moderately severe contamination for Cd [4]. The general contamination levels of tomatoes and green chilis are higher than that of cucumbers and eggplants, similar to the situation in greenhouses in Iran for tomatoes and cucumbers [55]. Different vegetable varieties have different pollutant absorption and transport capacities, but the soil pollution level and other resources are also comparative factors that cannot be ignored. For example, the vegetable AFs for the metals in Dianchi, southwestern China, were in the following order: Cd (0.0743) > Zn (0.0291) > Hg (0.0138) > Cu (0.0112) > As (0.0013) > Cr (0.0008) > Pb (0.0005) [65]; however, the concentrations of relative pollutants in the soil were much lower than that in this study.

### 3.3. Source Analysis of Target Heavy Metal(loid)s

The PCA results shown in Figure S4a and Tables S10 and S11 revealed that, for the soil samples, three factors explained 75.86% of the total variance, thereby indicating that the first three eigenvalues could represent the main contamination characteristics of the samples. The contribution of the first principal component (PC1) was 35.16%, and the characteristics of the factor variable values in all the target elements, except for Hg, were in positive load. The contribution of the second principal component (PC2) was 24.38%, and the characteristics of the factor variable values in Ni, As, Hg, and Cd were all in positive load. The contribution of the third principal component (PC3) was 16.32%, and the characteristics of the factor variable values in Zn, Hg, As, and Cu were all in positive load (Tables S11 and S12). The positive load of As in all three principal components indicated that As was the characteristic pollutant in the soil. The positions of the samples projected in the positive direction of the abscissa (PC1) in Figure S4a suggested that the greenhouse soils in Jingmen were mainly contaminated by Cu, Pb, and Cr, especially the green chili greenhouse in Guandang. Similarly, the positions of samples projected in the positive direction of the abscissa (PC2) suggests that the greenhouse soils in Jingmen were mainly contaminated by Zn, Hg, and As, especially the yellow cucumber and green eggplant greenhouses in Zilingpu. The position of samples projected in the positive direction of the abscissa (PC3) in Figure S4a suggests that the soil was mostly contaminated by Zn, Hg, As, and Cu,zespecially the samples from the Tuanlin greenhouse. In terms of regional differences, the primary typical pollutants in different soil samples were Ni and As for Zilingpu; Cu, Pb, and Cr for Zhanghe; Zn and Hg for Tuanlin; Cu, Pb, and Cr for Zhongxiang; Cr and Pb for Pengdun; Hg, Cr, and Pb for Xinshi; Hg for Yonglong; Ni and As for Lishi; and Cr, Cu, and Pb for Guandang.

**Figure 3:**
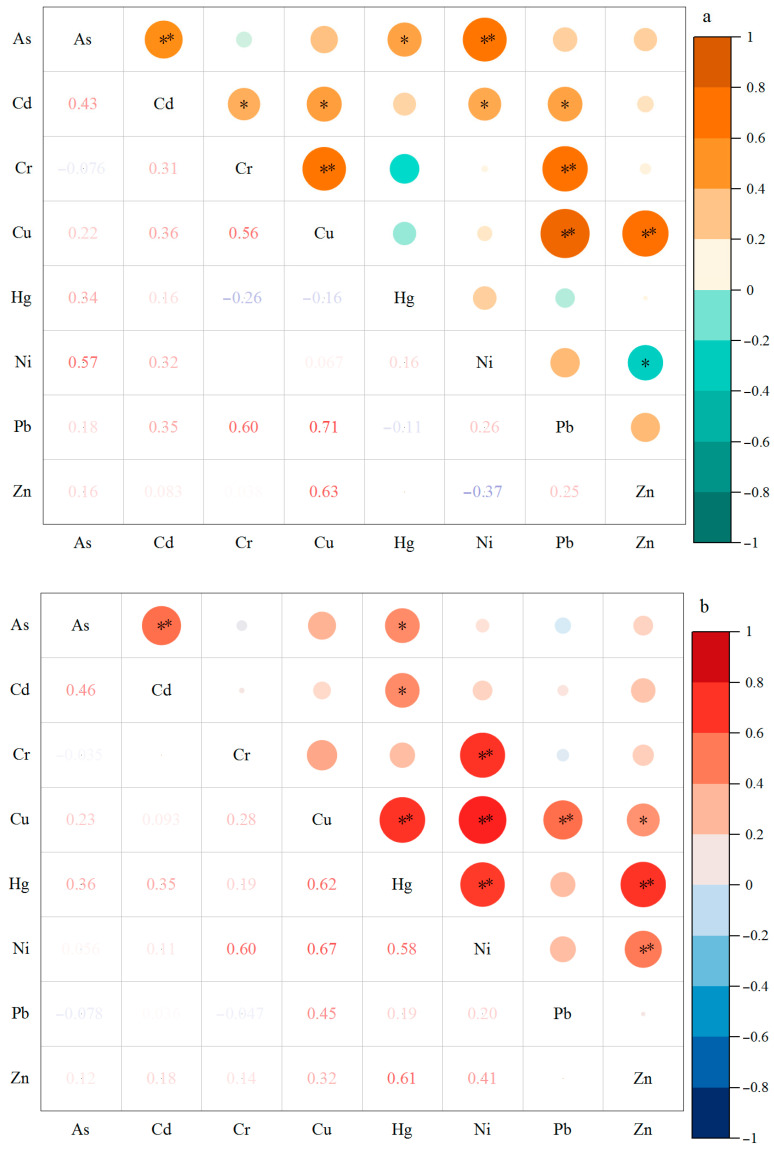

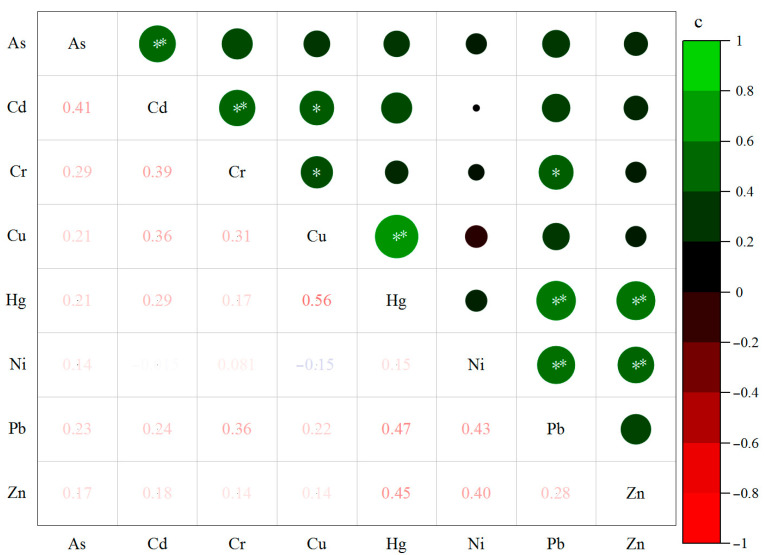
Correlation coefficients of target elements in different types of samples collected from the investigated greenhouses in Jingmen City. (**a**) Soil sample, (**b**) vegetable edible part sample, (**c**) vegetable leaf sample. * Indicates correlation is significant at *p* < 0.05 level; ** Indicates correlation is significant at *p* < 0.01 level.

Pearson correlation analyses of the target elements in the soil samples from the nine study areas in Jingmen are listed in Figure 3a. The correlations between As and Cd (0.01), Ni (0.01), and Hg (0.05) were significant. The correlations between Cd and Cr (0.05), Cu (0.05), Ni (0.05), and Pb (0.05) were significant. Cr and Cu had significant correlations with Cu (0.01) and Pb (0.01) and with Pb (0.01) and Zn (0.01), respectively. There was a significant correlation between Ni and Zn (0.05). Significant correlations indicate similar environmental sources or environmental behaviors between the elements or pollutants.

The PCA results shown in Figure S4b and Tables S10 and S11 indicate that, for the edible parts of vegetables, three factors explained 71.54% of the total variance, thereby indicating that the first three eigenvalues could represent the main contamination characteristics of all of the collected vegetable samples. The contribution of PC1 was 38.27%, and the characteristics of the factor variable values in all of the target elements were all in positive load, especially for Hg, Ni, and Cu. The contribution of PC2 was 18.79%, and the characteristics of the factor variable values in As, Cd, Hg, and Zn were all in positive load. The contribution of PC3 was 14.49%, and the characteristics of the factor variable values in Pb, Cu, and Hg were all in positive load (Tables S11 and S12). The positive load of Hg in all three principal components indicated that Hg was the characteristic pollutant in the edible parts of the vegetables. The positions of the samples projected in the positive direction of the abscissa (PC1) in Figure S4b suggested that the greenhouse vegetables in Jingmen were mainly contaminated by Hg, Ni, and Cu, especially those from the cherry tomato greenhouses in Pengdun and Guandang. The positions of the samples projected in the positive direction of the abscissa (PC2) suggested that the greenhouse soils in Jingmen were mainly contaminated by As, Cd, Hg, and Zn, especially the Chinese cabbage greenhouses of Zhongxiang and garland chrysanthemum greenhouses of Tuanlin. The position of samples projected in the positive direction of the abscissa (PC3) in Figure S4b suggested that the soil was mostly contaminated by Pb, Cu, and Hg, especially green chili from the greenhouses in Zhongxiang and Pengdun. In terms of regional differences, the primary typical pollutants in the edible vegetable samples were Hg and Ni for Zilingpu; Cr for Zhanghe; Cr and Ni for Tuanlin; Hg for Zhongxiang; Cu for Pengdun; Ni for Xinshi, Yonglong, and Lishi; and Pb and Cu for Guandang.

The PCA results shown in Figure S4c and Tables S10 and S11 indicate that, for vegetable leaves, three factors explained 67.07% of the total variance, thereby indicating that the first three eigenvalues could represent the main contamination characteristics of all of the collected vegetable leaf samples. The contribution of PC1 was 35.49%, and the characteristics of the factor variable values in all of the target elements were all in positive load, especially for Hg and Pb. The contribution of PC2 was 18.24%, and the characteristics of the factor variable values in Ni, Zn, Pb, and Hg were all in positive load. The contribution of PC3 was 13.33%, and the characteristics of the factor variable values in As, Cr, Cd, Ni, and Pb were all in positive load (Tables S11 and S12). The positive loads of Ni and Pb in all three principal components indicated that Ni and Pb were the characteristic pollutants in the vegetable leaves. The positions of the samples projected in the positive direction of the abscissa (PC1) in Figure S4c suggests that the vegetable leaves in the greenhouse of Jingmen were mainly contaminated by Hg and Pb, especially those from the Chinese tarragon and water spinach greenhouse in Lishi. The positions of the samples projected in the positive direction of the abscissa (PC2) suggest that the greenhouse soils in Jingmen were mainly contaminated by Ni, Zn, Pb, and Hg, especially in the green eggplant and yellow cucumber greenhouses of Zilingpu. The position of the samples projected in the positive direction of the abscissa (PC3) in Figure S4c suggests that the soil was mainly contaminated by As, Cr, Cd, Ni, and Pb, especially in the asparagus lettuce and Chinese cabbage greenhouse in Zhongxiang. In terms of regional differences, the primary typical pollutants in the different vegetable leaves were Ni in Zilingpu; Hg in Zhanghe and Tuanlin; Cd, Cr, and As in Zhongxiang; Zn and Hg in Pengdun; Cu in Xinshi, Yonglong, and Guandang; and Cd, Cr, and As in Lishi.

The Pearson correlation analysis results for the target elements in the edible vegetable parts and leaves from the nine study areas in Jingmen are listed in Figure 3b,c. In the edible vegetable parts, there were significant correlations between As and Cd (0.01) and Hg (0.05). Cd was significantly correlated with Hg (0.05). The correlations of Cr with Ni (0.01) and Cu with Hg (0.01), Ni (0.01), Pb (0.01), and Zn (0.05) were significant. The correlation between Hg, Ni, and Zn was 0.01. In the vegetable leaves (Figure 3c), there were significant correlations between As and Cd (0.01), Cd and Cr (0.01), Cu and Hg (0.01), Hg and Pb (0.01), Hg and Zn (0.01), Ni and Pb (0.01), and Ni and Zn (0.01). Significant correlations between Cd and Cu (0.05), Cr and Cu (0.05), and Cr and Pb (0.05) were also observed.

**Figure 4:**
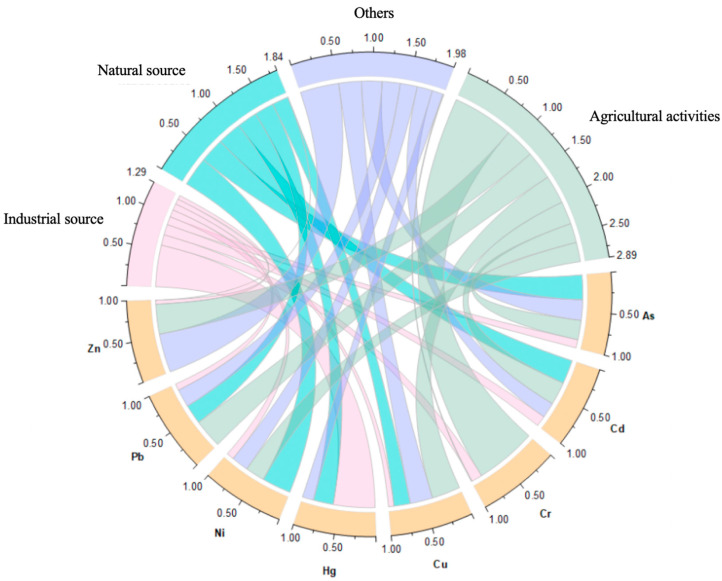
Chord diagram of source profiles and the contributions of target pollutants in the soils of investigated greenhouses in Jingmen from PMF.

The high homology between heavy metals in agricultural soils indicates that they may have common sources, such as lithogenic components, soil parent materials, and agricultural activities [66]. Based on published data regarding the source analysis of greenhouse soil pollutants, two main sources have been traced for the eight target pollutants, including anthropogenic sources (agricultural activities, industrial sources, and other sources) and natural sources (Figure 4). In general, the order is agricultural activities > other sources > natural sources > industrial sources, in this study (Figure 4). Fertilizers, especially livestock and poultry manure, are a major source of all eight target pollutants in the soils of greenhouses [6,7,50,52,67,68]. Overfertilization has caused major salinization and acidification in soils used for greenhouse vegetable production [69], and the stable increased organic matter contents in this study also constitute striking evidence (Table S1). Cd, Cu, Hg, Zn, Cr, Pb, and Ni accumulation and soil degradation in greenhouses can also occur due to chemical fertilizer application [70,71]. The use of different agricultural applications and downstream river erosion, river or sewage irrigation, and wind transportation may also affect heavy metal(loid) concentrations [72,73]. The use of As-contaminated water for irrigation results in a gradual accumulation of As in the topsoil and vegetables [74], although this was not clearly demonstrated in our study. The exposure concentrations of Cd were remarkably altered by anthropogenic activities, and many studies have confirmed that the use of chemical fertilizers, pesticides, and plastic products in agricultural production can lead to an increase in Cd in the soil [75]. In addition, pesticides and herbicides containing arsenide have been widely used in agricultural production [76], leading to the accumulation of As in soils after long-term use [77]. Anthropogenic sources of As in water include municipal, industrial, and domestic waste disposal, whereas coal combustion is a source of As pollution in different environmental matrixes [12]. Although most greenhouses are closed most of the time, pollution sources from outside can still affect the pollution levels inside. For example, fossil fuel combustion in industrial sources, automobile exhaust emissions, and even metal smelting in some mining areas can result in the contamination of Cd, Hg, Pb, and other pollutants in the soil through air circulation and atmospheric sedimentation [7,72,78]. Although the proportion of natural sources of pollutants is not excessive, it is still an important channel that cannot be ignored [50]. The Igeo of Ni in our study suggested that the change from the moderate to strong contamination level found in soils based on the old geochemical background values to the unpolluted level based on the updated geochemical background values was due to the increase in background values. A high geochemical background refers to soils that have accumulated a large quantity of Ni as a result of geogenic processes, including the weathering of parent materials and subsequent pedogenic processes [79]. Natural rock weathering can be a major contributor of Ni, accounting for approximately 60% [80]. Ni enrichment has been reported in soils developed from basalt and black shale parent materials [81], which are also important components of soil parent material across Hubei Province, from Enshi to Zaoyang and from Danjiangkou to Honghu [35]. However, this might not be the only reason for the increase in the geochemical background values of Ni. Source analysis based on long-term location accumulation data monitoring are promising future directions to elucidate this.

It has been found that the accumulations and bioavailability of Cd, Cu, and Zn were severe in plastic shed production systems, and the risks from tomato-based rotations were higher than others, indicating a greater degree of heavy metal accumulation in tomatoes [2]. In Figure S1, the accumulative concentrations of all target pollutants in cherry tomatoes from Pengdun is the highest among all four of the most commonly planted vegetables (over 80 mg/kg). In other studies, leafy vegetables that contain higher concentrations of heavy metals than fresh fruits and fruit vegetables have been detected [82], while in this study, the accumulative concentrations of the eight target pollutants were the highest for Chinese cabbage (mean 89.47 mg/kg) (Table S7). It has also been proven that the order of heavy metal uptake by vegetables (Zn > Pb > Cr > As > Ni > Cd > Cu > Hg in edible parts; As > Zn > Cr > Pb > Ni > Cd > Cu > Hg in leaves) is not necessarily in the same order as those in soils (Zn > Cr >Ni > Pb > As > Cd > Cu > Hg), because soil contamination is not the only pollution source [55]. Both the contents and the PCA results showed that the Hg concentration was low and below the contaminant limit in soils, whereas that in most vegetables was higher than the contaminant limit; therefore, contamination might have originated from extraneous sources. For example, fertilizers often contain elevated amounts of Hg as an impurity, and Hg in drinking water is also an important source [83].

### 3.4. Human Health Risks

**Figure 5:**
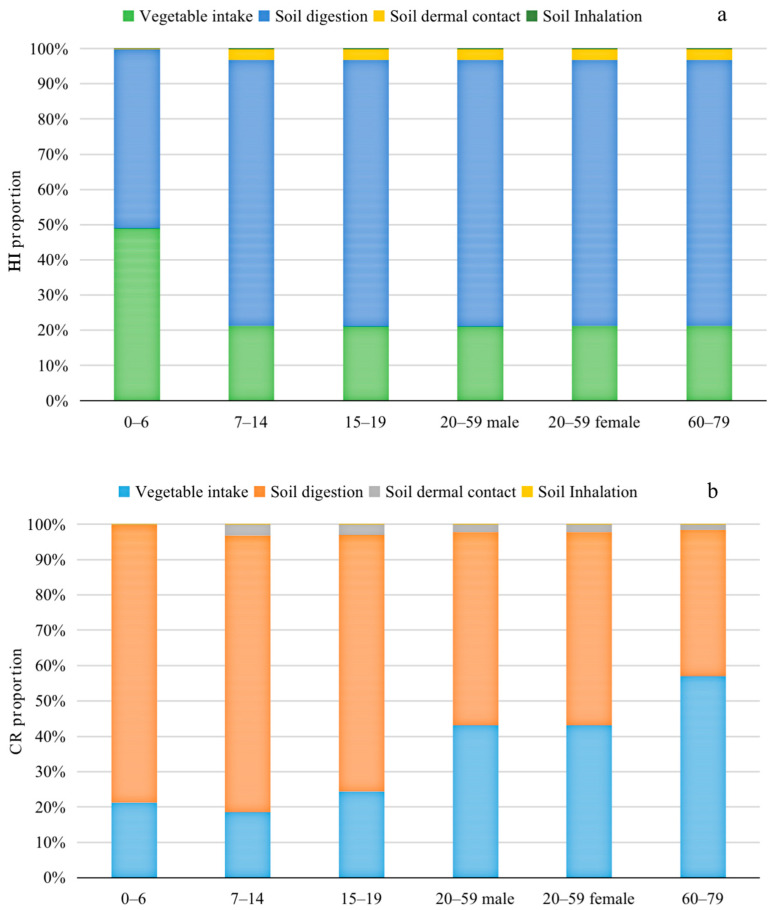
Contribution of different exposure routes to non-carcinogenic and carcinogenic risks. (**a**) HI proportion for different exposure routes in different age groups; (**b**) CR proportion for different exposure routes in different age groups. Refer to Figure S1 for the denoted values.

Risk assessment evaluates the potential health effects of a contaminant through one or more exposure pathways on humans [50]. Heatmaps of non-CRs based on the HI values from collected vegetable samples to vegetable consumers and from soil samples to greenhouse workers in the nine study areas are shown in Figure S5. The non-carcinogenic health risks posed by the eight target elements from different pathways varied considerably in the order of ingestion > intake > dermal > inhalation, but no HQ values were higher than 1 (Figure 5). For HQ values among different age groups in this study, the non-CR values for children aged 0 to 6 years were relatively higher than for the other groups (Figure S5), although non-carcinogenic health risks were posed by any of the eight target heavy metal(loid)s to either vegetable consumers or greenhouse workers. In this study, the classification of different age groups also determined the precision of the result. For example, adults aged between 20 and 59 could have been divided into younger (20–44) and middle-aged (45–59) adults, but parameters other than body weight (65.39 kg and 66.12 kg, respectively) [41] had little influence on the calculation process, and the weight difference between the two groups was not large, so the division was of little significance. However, for adolescents, children aged between 7 and 14 (36.4 kg) exhibited approximately half the body weight of children aged between 15 and 19 (68.75 kg), so division by age group was of more importance here. On the other hand, the average body weights of the different age groups should also be corrected, as the development of living standards has an effect on the average weight during the assessment period. Moreover, there is variation in the areas assessed (e.g., different countries), which is why the assessment of contamination and health risks should be called a living science. The results obtained from farmland soils were more reassuring than those from mining areas, because the HQs in mining areas were often reported to be greater than 1 for different heavy metal(loid)s [12,84]. Even in the vicinity of contaminated sites in Guangdong Province, the health risk to the surrounding residents has been proven to be relatively low from Cu and Zn uptake via the vegetable pathway, which was in agreement with our conclusion [84].

**Figure 6:**
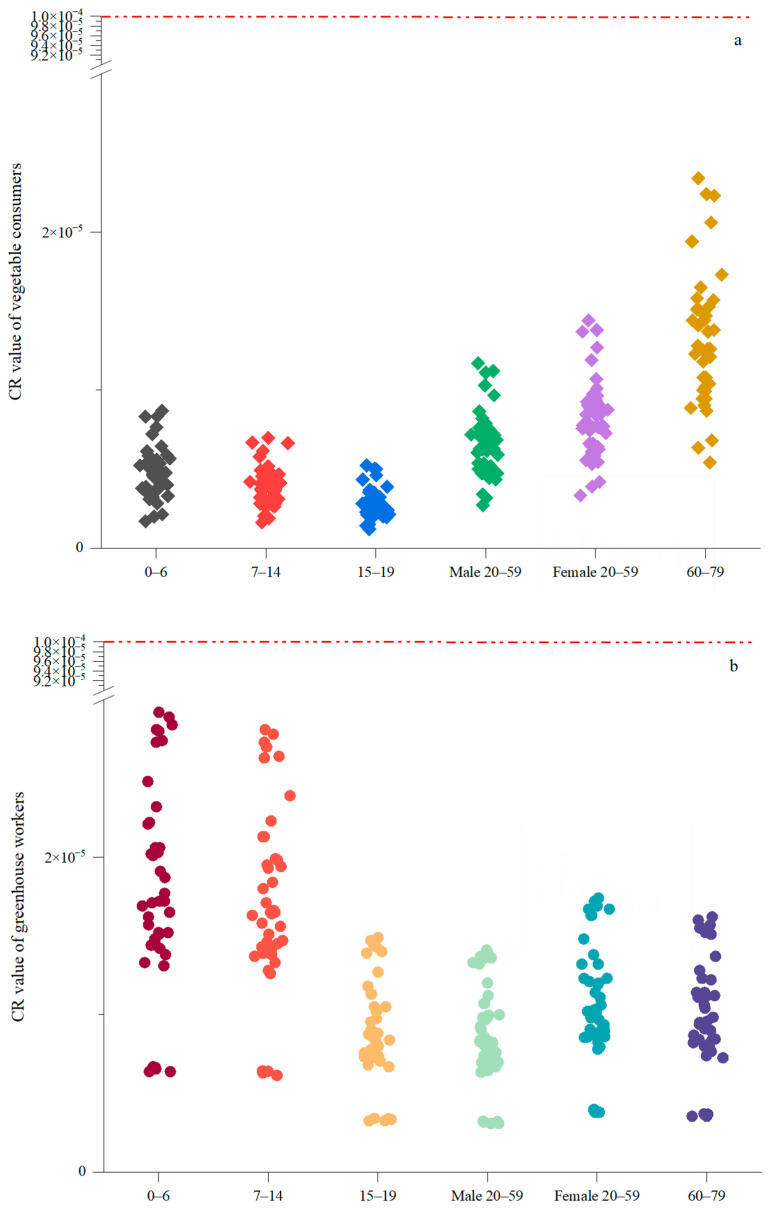

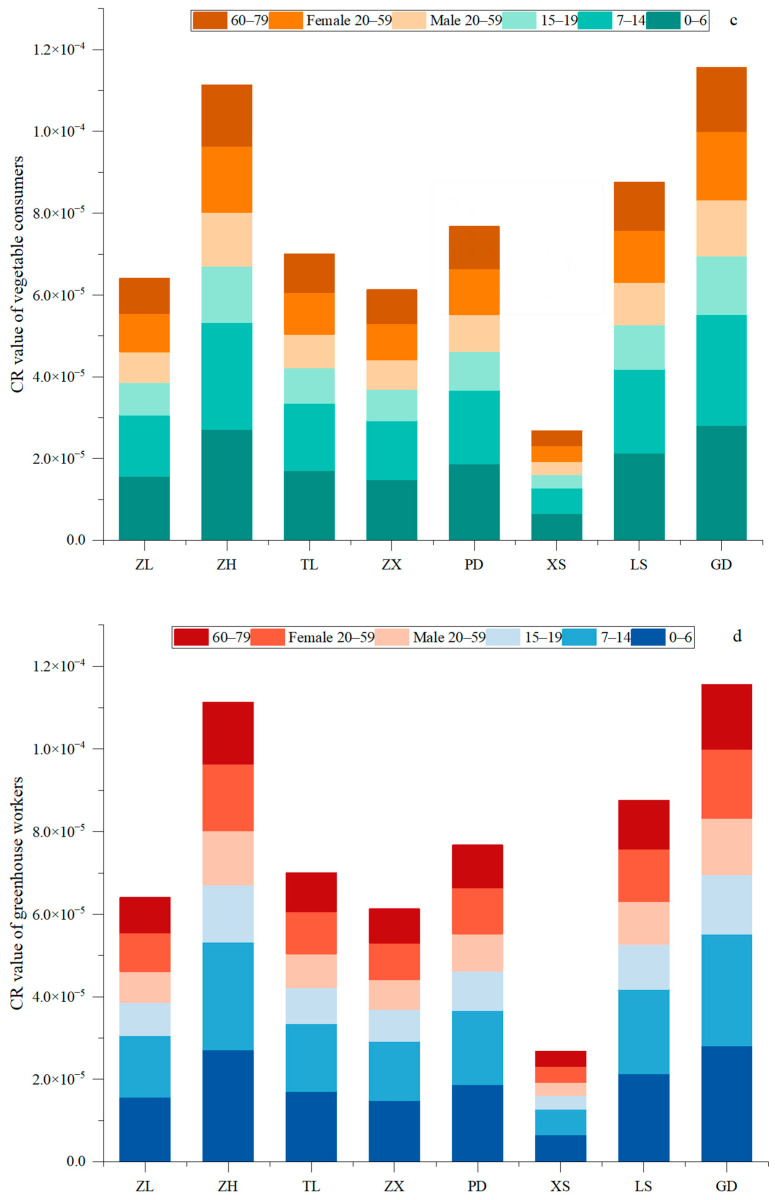
Comparison of carcinogenic risk (CR) values for (**a**) different age groups for all vegetable consumers, (**b**) different age groups for all investigated greenhouses workers, (**c**) consumers eating vegetables from the nine study areas in Jingmen City, and (**d**) workers from greenhouses in the nine study areas in Jingmen City. The red dash lines in (**a**,**b**) are the warning level of CR values as 1.0 × 10^−4^.

The threshold CR values for the different greenhouse sampling sites in Jingmen all lie between 10^−6^ and 10^−4^ in all age groups for both vegetable consumers (Figure 6a) and greenhouse workers (Figure 6b), indicating that the carcinogenic risks are all considered acceptable. For the different study areas, the carcinogenic health risks were the highest in Guandang and Zhanghe and lowest in Xinshi (Figure 6c,d). However, owing to the lack of an oral slope factor of the carcinogens, Cd, Cu, Hg, and Zn were not factored into the CR calculation. The contributions of the different toxic elements to the CR values of vegetable consumers were generally in the order of As > Ni > Cr > Pb for the different age groups, but for the greenhouse workers, these were generally in the order of Ni > As > Cr > Pb > Cd (Figure S6). In other studies, although the Cd and Cr concentrations in some pak choi samples exceeded the national thresholds, the non-CRs and CRs for both adults and children in the soil–pak choi system were acceptable, and remediation measures were also implemented [85]. A previous study found that the CR probabilities for Pb and Cr in children and adults were below the acceptable level in surface-exposed lawn soils from 28 urban parks in Guangzhou [86], which indicated that greenhouse production introduces more pollutants than lawn soil. The complexity of the diet also affects the assessment of CR. In farmland in Chile, where the As contamination level in vegetable edible parts is generally lower than that in Jingmen (max 4.0 mg/kg), the estimated CR (0.0012) value from vegetable consumption for children under 5 years old is unacceptable by means of 13 types of vegetables [87].

In another study, when HQs and CRs to human health were estimated, children had a higher risk via inhalation and dermal contact [88]. However, in our investigation, the contact time between young children and soil, mainly through recreation, was modified to one-third of that of adults because there was less contact by children with greenhouses than open fields. Nevertheless, this modification did not bring the CR to children much lower than that of older children and adults (Figure 6b). This agrees with the conclusion that trace metal accumulation and contamination in greenhouse soils may pose significant health risks to children via oral ingestion, inhalation, and dermal contact when playing in greenhouse fields [78]. Meanwhile, the uncertainty of health risk assessment should also be considered, as the concentration of some heavy metals may not only be increased by conventional cooking [89], but metals of different valence states and forms can also cause various harmful effects. In addition, certain assumptions made in exposure modeling, such as constant intake rates, uniform vegetable types, and the same cooking style, may result in significant inaccuracies. The different intake habits of raw, cooked, pickled, and fermented vegetables may contribute to fluctuations in pollutant concentrations. For example, the content of As, Pb, and Cd in leaf pickles was significantly higher than that in pickles of other types of vegetables [90]. Moreover, potential overestimation or underestimation might have also occurred due to bioavailability or cumulative exposure effects. In the future, in addition to the more precise modification of the exposure models, more comprehensive studies will be conducted on the migration and transport mechanisms of heavy metal(loid)s in different plant tissues and species and their associations with human health risks [91].

## 4. Conclusions

The pollution characteristics, ecological risks, and potential health risks of eight heavy metal(loid)s in nine typical greenhouse soils and vegetables in Jingmen, Hubei Province, Central China, were systematically investigated. Compared with the Chinese limitation values or guidelines for soil heavy metal(loid)s, the exceeding rates were 100% for Cd (2.9 to 22.1 times) and 9.09% for Ni. Compared with the Japanese guidelines and the Canadian Soil Quality Guidelines, the exceeding rates were 100% and 86.36% for Cd, 20.45% and 81.82% for As, 0 and 20.45% for Cr, and 0 and 2.27% for Ni, respectively. Cadmium was the element with the most prominent exceeding rate, followed by As, Cr, and Ni. The single pollution index of Cd was the highest, followed by As and Ni. The Nemerow integrated pollution indices for Lishi, Guandang, Zhanghe, Zilingpu, and Tuanlin were moderate, while the others were found to have low pollution levels. The ecological risks of the target Cd were 45.45% at the disastrous level and 54.55% at the high risk level, followed by 15.91% of Hg at the high risk level. The potential ecological risk indices for Zhanghe and Lishi indicated considerable to high risk levels. In the vegetable edible parts, 100% of As, Cd, Cr, Ni, and Pb and 40.91% of Hg (18/44) exceeded the standard values of Chinese Maximum Levels of Contaminants, the WHO and FAO permission limits, and the food recommendation limits set by the EU. The AFs of As and Cd were higher than those of the other metals.

Multivariate PCA indicated significant contributions of Cu, Pb, and Cr in the soil; Hg, Ni, and Cu in edible vegetable parts; and Hg, Pb, and Cd in vegetable leaves. The contributions to both non-carcinogenic and carcinogenic health risks via soil ingestion were the greatest, although none of these risks in either vegetable consumers or greenhouse workers exceeded the threshold values. However, the carcinogenic risks from vegetable consumption in older people (60–79 years old) and the carcinogenic risks from recreation in children of greenhouse workers (under 15 years old) should be given more consideration, especially in GD and ZH. Long-term greenhouse production periods have resulted in considerable contamination in the vegetable–soil systems of the nine study areas that should not be ignored. Officially recommended organic fertilizers and pesticides with standardized production should be applied to reduce Cd accumulation via fertilizers and other contamination from agricultural sources. Updating soil quality standards across China using local background values is vital to reduce misunderstandings regarding pollution and risk assessment. Moreover, soil remediation and risk mitigation strategies in high-risk areas such as Zhanghe and Lishi should also be implemented. Standardized production methods and clean production management can ensure the sustainable development of greenhouse agriculture and the safety of greenhouse agricultural products.

## Figures and Tables

**Table 1:** Natural background concentration of the target elements (mg/kg).

Study Area	Site	As	Cd	Cr	Cu	Hg	Ni	Pb	Zn
Hubei Province [34]	1–9	12.3	0.172	86.0	30.7	0.0800	3.73	26.7	83.6
Jingmen Central Area [35]	1–3	12.5	0.190	78.0	27.1	0.0571	33.40	31.0	67.0
Zhongxiang City [35]	4, 5	12.1	0.260	81.0	30.5	0.0515	37.50	27.5	82.0
Jingshan City [35]	6, 7	12.3	0.200	79.0	29.2	0.0630	34.50	29.9	73.0
Shayang County [35]	8, 9	12.1	0.150	76.0	24.9	0.0636	30.70	29.5	55.0

**Table 2:** Concentrations of target elements in the soils of greenhouses in Jingmen City (the mean ± SD) (dry weight/DW) ^1^.

No.	Area	Vegetable Type		As (mg/kg)	Cd (mg/kg)	Cr (mg/kg)	Cu (µg/kg)	Hg (µg/kg)	Ni (mg/kg)	Pb (mg/kg)	Zn (mg/kg)
1	Zilingpu (ZL) *n* = 24	Green eggplant		15.06 ± 0.16	***2.53 ± 0.55***	17.91 ± 1.29	152.98 ± 3.88	87.43 ± 3.47	**38.66 ± 5.00**	20.55 ± 0.64	73.05 ± 4.16
		Yellow cucumber		15.95 ± 0.21	***2.07 ± 0.90***	11.16 ± 3.00	144.34 ± 7.11	97.15 ± 3.03	**35.65 ± 6.61**	17.56 ± 0.58	72.02 ± 1.38
		Cucumber		13.92 ± 0.14	***2.51 ± 0.83***	12.20 ± 3.10	146.13 ± 5.08	50.06 ± 2.21	**37.26 ± 5.51**	26.16 ± 0.53	72.47 ± 0.90
		Tomato		13.44 ± 0.70	***1.55 ± 0.50***	32.48 ± 2.27	167.90 ± 4.63	28.17 ± 0.20	**37.80 ± 5.21**	17.19 ± 0.75	81.17 ± 2.38
		Green chili		11.23 ± 0.12	***2.09 ± 0.78***	23.64 ± 2.58	178.29 ± 2.27	70.04 ± 3.45	31.36 ± 6.05	19.43 ± 0.87	73.21 ± 5.46
		Small cushaw		14.00 ± 0.90	***1.73 ± 0.35***	64.47 ± 3.95	164.37 ± 5.65	58.71 ± 1.72	33.40 ± 3.76	22.05 ± 1.41	72.39 ± 5.19
2	Zhanghe (ZH) *n* = 12	Long purple eggplant		13.43 ± 0.96	***2.53 ± 1.13***	75.39 ± 2.48	188.17 ± 13.70	26.32 ± 0.57	28.03 ± 2.26	22.05 ± 1.97	124.90 ± 5.53
		Green chili		11.91 ± 0.40	***2.11 ± 0.43***	66.29 ± 1.59	205.76 ± 8.48	20.65 ± 0.34	27.06 ± 3.63	22.05 ± 2.27	168.02 ± 1.39
		Cauliflower		12.73 ± 0.90	***3.42 ± 3.21***	81.12 ± 2.41	196.07 ± 7.63	59.41 ± 1.39	32.65 ± 2.60	22.42 ± 2.23	114.03 ± 9.71
3	Tuanlin (TL) *n* = 24	Cherry tomato		13.62 ± 0.56	***1.73 ± 0.13***	16.89 ± 2.03	175.26 ± 10.58	58.72 ± 0.37	30.07 ± 7.63	15.70 ± 0.58	163.54 ± 5.20
		Long purple eggplant		13.67 ± 0.50	***2.69 ± 0.83***	11.42 ± 1.49	173.70 ± 10.57	38.85 ± 2.36	29.21 ± 4.05	16.82 ± 0.40	159.10 ± 3.92
		Pumpkin		13.94 ± 0.60	***1.51 ± 0.10***	12.73 ± 1.99	202.59 ± 7.58	46.57 ± 2.33	30.71 ± 2.98	18.31 ± 0.06	194.82 ± 3.61
		Cucumber		14.18 ± 0.12	***1.58 ± 0.47***	12.73 ± 1.17	174.52 ± 11.95	93.17 ± 1.91	31.57 ± 3.80	19.43 ± 1.34	170.72 ± 6.21
		Lettuce		15.39 ± 0.88	***1.78 ± 0.89***	10.12 ± 1.70	176.00 ± 8.02	22.79 ± 0.53	32.32 ± 4.37	22.05 ± 0.17	151.92 ± 0.76
		Garland chrysanthemum		15.34 ± 0.60	***1.73 ± 0.35***	11.42 ± 1.19	223.86 ± 9.53	42.70 ± 0.34	29.21 ± 5.49	25.04 ± 0.90	195.97 ± 2.18
4	Zhongxiang (ZX) *n* = 16	Cauliflower		11.14 ± 0.83	***1.75 ± 0.37***	66.02 ± 5.57	206.45 ± 9.47	31.07 ± 1.63	25.13 ± 5.59	26.53 ± 2.07	141.78 ± 7.56
		Asparagus lettuce		12.50 ± 0.34	***2.95 ± 2.02***	36.37 ± 1.88	162.35 ± 14.71	16.47 ± 0.59	32.22 ± 6.47	21.30 ± 1.25	83.39 ± 5.34
		Chinese cabbage		12.10 ± 0.55	***1.71 ± 0.27***	73.57 ± 2.11	178.84 ± 9.86	28.57 ± 0.71	29.42 ± 5.00	24.29 ± 2.17	88.75 ± 4.25
		Green chili		10.09 ± 0.49	***1.89 ± 0.47***	33.25 ± 5.19	161.06 ± 16.33	42.93 ± 0.83	32.43 ± 6.22	19.06 ± 1.14	77.64 ± 4.58
5	Pengdun (PD) *n* = 16	Cherry tomato		12.63 ± 0.64	**1.18 ± 0.04**	28.07 ± 4.23	184.12 ± 6.34	38.64 ± 0.15	34.58 ± 4.89	26.16 ± 1.24	102.01 ± 7.67
		Mini cucumber		13.21 ± 0.12	***1.73 ± 0.77***	80.08 ± 2.11	193.72 ± 10.22	31.75 ± 1.63	35.22 ± 3.78	25.78 ± 1.76	101.74 ± 4.83
		Purple eggplant		13.21 ± 0.12	***1.44 ± 0.38***	83.20 ± 1.87	182.33 ± 12.52	29.65 ± 1.28	33.93 ± 3.29	24.29 ± 1.08	96.09 ± 2.54
		Green chili		12.37 ± 0.25	***1.84 ± 0.50***	64.74 ± 2.87	207.69 ± 10.83	24.62 ± 0.66	35.33 ± 4.99	22.05 ± 1.30	96.84 ± 3.04
6	Xinshi (XS) *n* = 20	Cucumber		10.28 ± 0.16	**1.18 ± 0.17**	13.33 ± 3.05	137.95 ± 8.63	22.03 ± 0.46	30.59 ± 3.68	17.27 ± 0.86	73.20 ± 2.97
		Tomato		10.83 ± 0.14	***1.28 ± 0.26***	12.39 ± 2.84	133.67 ± 6.32	23.55 ± 0.52	31.55 ± 5.17	16.38 ± 0.65	71.95 ± 3.85
		Bitter gourd		10.35 ± 0.13	**1.12 ± 0.25**	12.34 ± 1.69	146.85 ± 5.95	20.98 ± 0.32	30.98 ± 4.57	15.40 ± 1.24	73.16 ± 4.67
		Purple eggplant		10.97 ± 0.02	***1.30 ± 0.52***	15.40 ± 3.55	144.40 ± 7.66	22.01 ± 0.49	30.68 ± 3.68	14.96 ± 0.95	74.68 ± 3.88
		Green chili		11.03 ± 0.31	***1.35 ± 0.35***	14.68 ± 2.28	146.35 ± 8.18	22.84 ± 0.52	31.23 ± 3.82	15.85 ± 0.84	74.62 ± 5.85
7	Yonglong (YL) *n* = 20	Tomato		14.24 ± 0.51	***1.66 ± 0.27***	25.65 ± 2.23	158.62 ± 6.65	52.35 ± 0.62	33.95 ± 3.45	18.64 ± 0.96	69.16 ± 4.65
		Green chili		13.56 ± 0.15	***1.67 ± 0.38***	22.39 ± 3.82	146.63 ± 6.95	51.00 ± 0.31	32.63 ± 3.69	19.65 ± 0.55	83.96 ± 4.95
		Long purple eggplant		13.65 ± 0.22	***1.78 ± 0.42***	25.95 ± 4.15	155.33 ± 5.85	52.49 ± 0.29	31.06 ± 5.17	18.65 ± 0.67	88.19 ± 3.48
		Cucumber		14.02 ± 0.32	***1.75 ± 0.32***	26.38 ± 3.19	153.32 ± 8.15	55.15 ± 0.32	33.18 ± 4.98	19.85 ± 0.79	82.17 ± 3.85
		Cowpea		12.35 ± 0.22	***1.69 ± 0.52***	22.95 ± 2.95	159.32 ± 7.56	52.15 ± 0.42	32.02 ± 4.64	18.31 ± 0.95	78.17 ± 3.95
8	Lishi (LS) *n* = 24	Cucumber		15.63 ± 0.30	***2.14 ± 0.72***	36.65 ± 2.84	186.35 ± 8.33	38.15 ± 0.32	43.02 ± 4.97	22.32 ± 1.36	85.14 ± 4.84
		Chinese tarragon		17.69 ± 0.33	***2.92 ± 0.56***	39.65 ± 2.62	188.34 ± 4.96	39.62 ± 0.28	41.94 ± 3.11	23.54 ± 1.27	95.61 ± 5.19
		Water spinach		17.33 ± 0.17	***2.87 ± 0.56***	39.69 ± 3.42	195.65 ± 8.67	42.49 ± 0.34	38.29 ± 2.87	20.52 ± 1.10	98.62 ± 2.94
		White tomato eggplant		15.55 ± 0.33	***2.19 ± 0.57***	33.85 ± 3.85	187.33 ± 9.16	33.15 ± 0.38	36.19 ± 5.85	21.63 ± 1.38	86.62 ± 8.81
		Green chili		16.32 ± 0.35	***1.97 ± 0.84***	35.17 ± 2.94	176.34 ± 6.32	33.28 ± 0.52	35.17 ± 3.17	20.65 ± 1.39	88.85 ± 7.65
		Tomato		14.33 ± 0.22	***2.00 ± 0.74***	35.30 ± 4.89	179.68 ± 5.95	36.48 ± 0.94	38.17 ± 5.86	21.08 ± 1.52	87.61 ± 6.62
9	Guandang (GD) *n* = 20	Mini cucumber		12.39 ± 0.22	***2.32 ± 0.62***	55.32 ± 3.19	210.32 ± 8.12	38.32 ± 0.65	35.67 ± 3.16	25.62 ± 0.86	85.61 ± 4.59
		Cherry tomato		13.26 ± 0.22	***2.57 ± 0.59***	52.32 ± 2.92	209.32 ± 5.96	32.62 ± 0.91	33.69 ± 3.25	26.65 ± 0.95	110.27± 5.89
		Broccoli		12.99 ± 0.32	***2.16 ± 0.62***	52.85 ± 2.88	207.96 ± 6.68	40.62 ± 0.88	35.85 ± 3.40	28.17 ± 0.86	129.17 ± 4.95
		Purple ball eggplant		13.28 ± 0.62	***2.13 ± 0.66***	53.23 ± 2.62	205.36 ± 5.69	38.17 ± 1.06	36.18 ± 5.52	27.62 ± 0.86	135.05 ± 6.85
		Green chili		13.33 ± 0.59	***2.37 ± 0.75***	54.22 ± 2.34	220.62 ± 5.98	36.66 ± 1.10	37.17 ± 4.97	29.62 ± 0.86	143.49 ± 5.92
GB 15618-2018 [46]			pH ≤ 5.5	40	0.3	150	50,000	1300	60	70	200
			5.5 < pH ≤ 6.5	40	0.3	150	50,000	1800	70	90	200
			6.5 < pH ≤ 7.5	30	0.3	200	100,000	2400	100	120	250
HJ/T 333-2006 [9]			pH < 6.5	30	0.3	150	50,000	250	40	50	200
			6.5 < pH < 7.5	25	0.3	200	100,000	300	50	50	250
			7.5 < pH	20	0.4	250	100,000	350	60	50	300
NY/T 391-2021 [10]			pH < 6.5	25	0.3	120	50,000	250	/	50	/
			6.5 < pH < 7.5	20	0.3	120	60,000	300	/	50	/
			7.5 < pH	20	0.4	120	60,000	350	/	50	/
Japanese guidelines [47]				15	0.4	/	125,000	/	/	/	/
Canadian guidelines [48]				12 (1997)	1.4 (1999)	64 (1997)	63,000 (1999)	6600 (1999)	45 (2015)	70 (1999)	250 (2018)

^1^ “/” denotes not listed; bold numbers indicate exceeding the Chinese limits; underlined numbers indicate exceeding the international suggested values or guidelines; italic bold numbers indicate exceeding both the domestic and the international suggested values or guidelines.

## Data Availability

The data presented in this study are available upon request from the corresponding author.

## Supplementary Materials

The following supporting information can be downloaded at: https://www.mdpi.com/article/10.3390/toxics13040312/s1, Figure S1: Accumulation concentrations of the eight target elements in soils and vegetable parts of the four most frequently planted vegetables (mg/kg); Figure S2: Geo-accumulation index (Igeo) of the eight target pollutants in investigated greenhouses of Jingmen (*n* = 176); Figure S3: Accumulation factors (AFs) of the eight target pollutants in vegetable edible parts from investigated greenhouses of Jingmen (*n* = 176); Figure S4: Principal component analysis (PCA) of the eight target elements in samples collected from nine study areas; Figure S5: Heatmap of non-carcinogenic risks based on the HI values from the collected samples in the nine study areas; Figure S6: Contributions of different elements to the carcinogenic risks of people in different age groups; Table S1: Physico-chemical characteristic of soils and overview of production information in the nine study areas; Table S2: Classification of baseline values for the Igeo, PI, NIPI, Er and RI; Table S3: Parameters for health risk assessment; Table S4: Nemerow integrated pollution index and the risk level assessment results of target elements in the greenhouses of the nine study areas of Jingmen City; Table S5: Er values of the target elements in the greenhouses of the nine study areas in Jingmen City; Table S6: RI values and the risk level assessment results of the target elements in greenhouses of the nine study areas of Jingmen City; Table S7: Concentrations of the target elements in vegetable edible parts of greenhouses in Jingmen City (mean ± SD) (dry weight/DW); Table S8: Concentrations of target elements in the vegetable leaves of greenhouses in Jingmen City (mean ± SD) (dry weight/DW); Table S9: Accumulation factor (AF) of target elements in vegetables of greenhouses in Jingmen City (mean ± SD); Table S10: Principal component analysis (PCA) of the eight target elements in different samples collected from the nine study areas in Jingmen City; Table S11: Characteristic value and accumulative contribution of the PCA.
