# Supplementary material for: Ecological Risk Assessment Is a Living Science: A Study on Heavy Metal(loid) Contamination in Typical Greenhouse Production Systems in Central China
Source: Toxics. 2025 Apr 17;13(4):312. doi: 10.3390/toxics13040312 (PMC12031059; doi:10.3390/toxics13040312)
Supplement: Supplementary file 1 [file toxics-13-00312-s001.zip › toxics-3558493-supplementary.pdf]

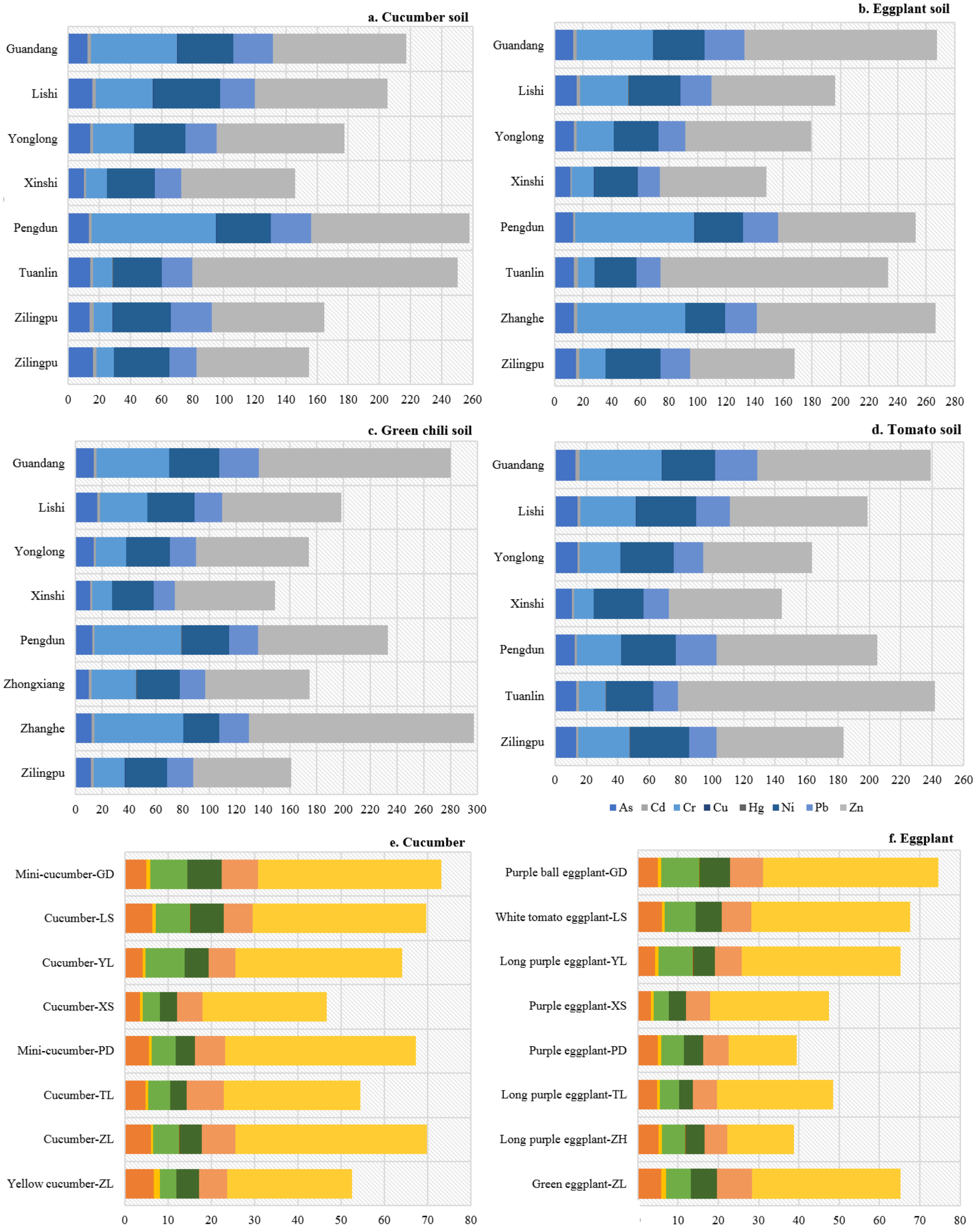
a. Cucumber soil
Guandang
Lishi
Yonglong
Xinshi
Pengdun
Tuanlin
Zilingpu
Zilingpu
0 20 40 60 80 100 120 140 160 180 200 220 240 260
b. Eggplant soil
Guandang
Lishi
Yonglong
Xinshi
Pengdun
Tuanlin
Zhanghe
Zilingpu
0 20 40 60 80 100 120 140 160 180 200 220 240 260 280
c. Green chili soil
Guandang
Lishi
Yonglong
Xinshi
Pengdun
Zhongxiang
Zhanghe
Zilingpu
0 20 40 60 80 100 120 140 160 180 200 220 240 260 280 300
d. Tomato soil
Guandang
Lishi
Yonglong
Xinshi
Pengdun
Tuanlin
Zilingpu
0 20 40 60 80 100 120 140 160 180 200 220 240 260
As Cd Cr Cu Hg Ni Pb Zn
e. Cucumber
Mini-cucumber-GD
Cucumber-LS
Cucumber-YL
Cucumber-XS
Mini-cucumber-PD
Cucumber-TL
Cucumber-ZL
Yellow cucumber-ZL
0 10 20 30 40 50 60 70 80
f. Eggplant
Purple ball eggplant-GD
White tomato eggplant-LS
Long purple eggplant-YL
Purple eggplant-XS
Purple eggplant-PD
Long purple eggplant-TL
Long purple eggplant-ZH
Green eggplant-ZL
10 20 30 40 50 60 70 80

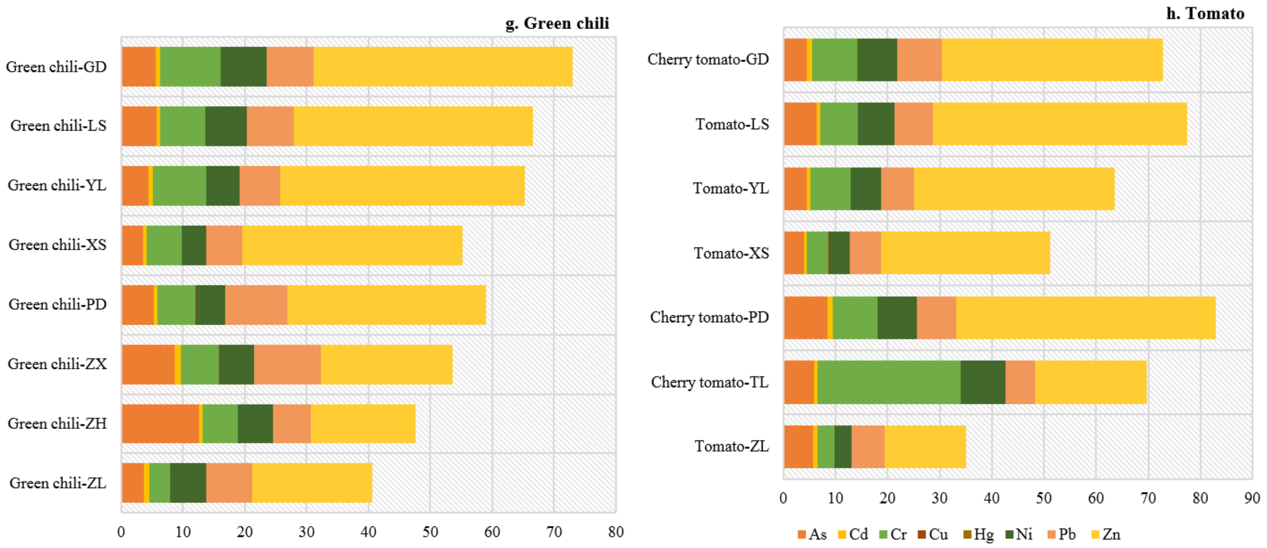

Figure S1. Accumulation concentrations of eight target elements in soils and vegetable parts of the four most frequently planted vegetables (mg/kg). ZL, Zilingpu; ZH, Zhanghe; TL, Tuanlin; ZX, Zhongxiang; PD, Pengdun; XS, Xinshi; YL, Yonglong; LS, Lishi; GD, Guandang.

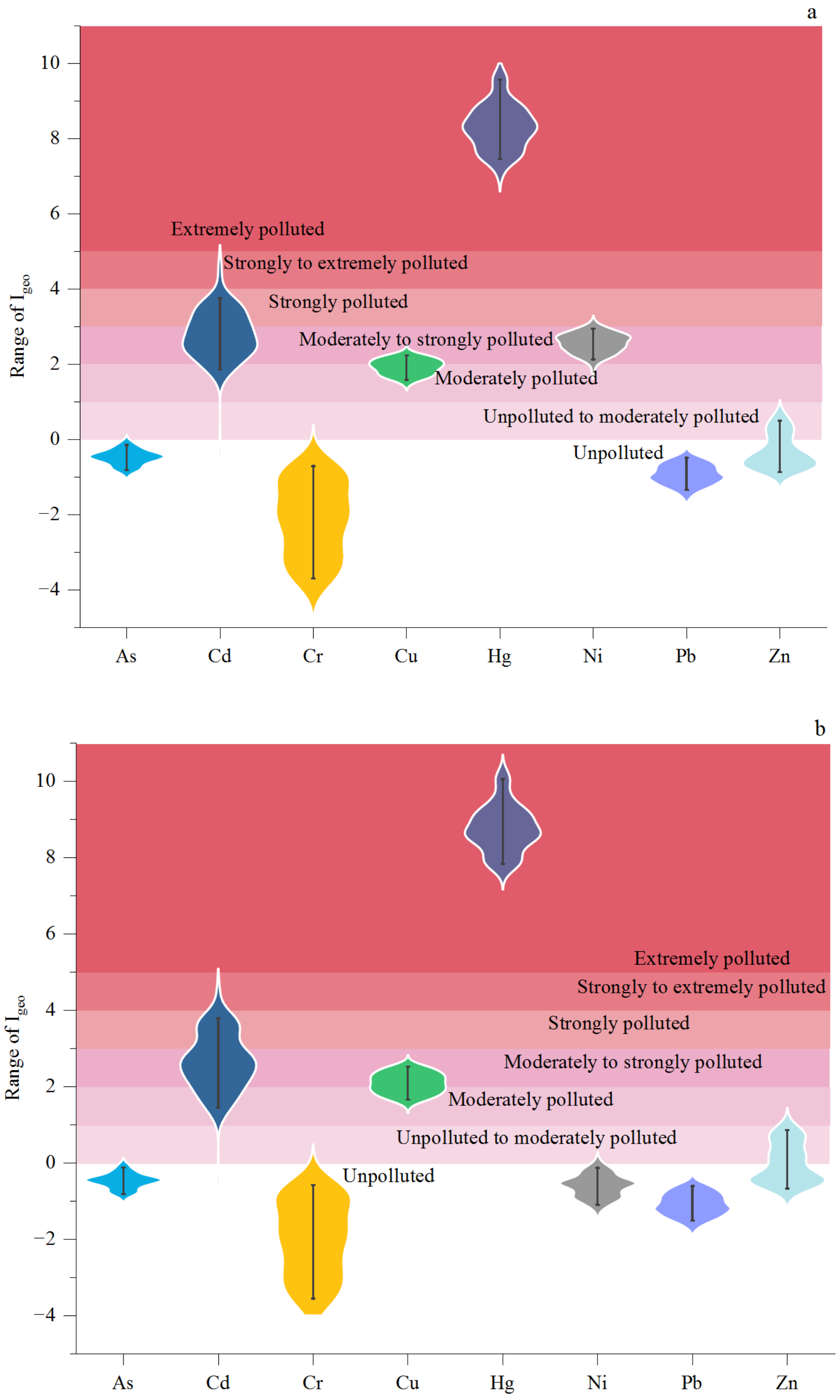

Figure S2. Geo-accumulation index (Igeo) of eight target pollutants in investigated greenhouses of Jingmen (n=176). a) Igeo values calculated on 1990 version background values [34]; b) Igeo values calculated on 2023 version background values [35]. Error bar indicates data between 5% and 95%.

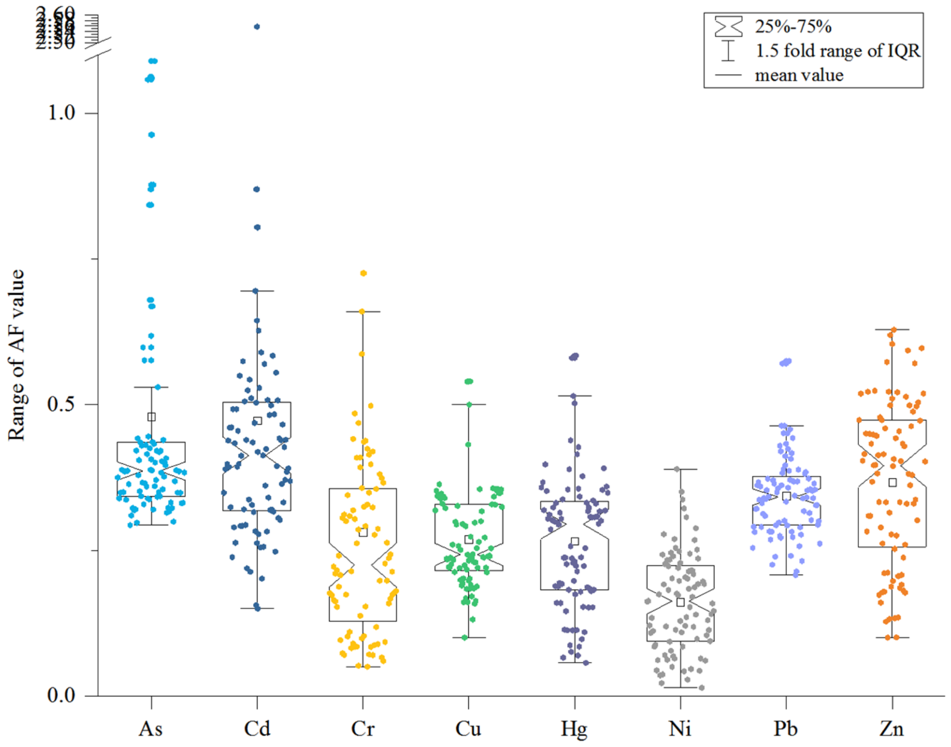

Figure S3. Accumulation factors (AFs) of the eight target pollutants in vegetable edible parts from investigated greenhouses of Jingmen (n=176).

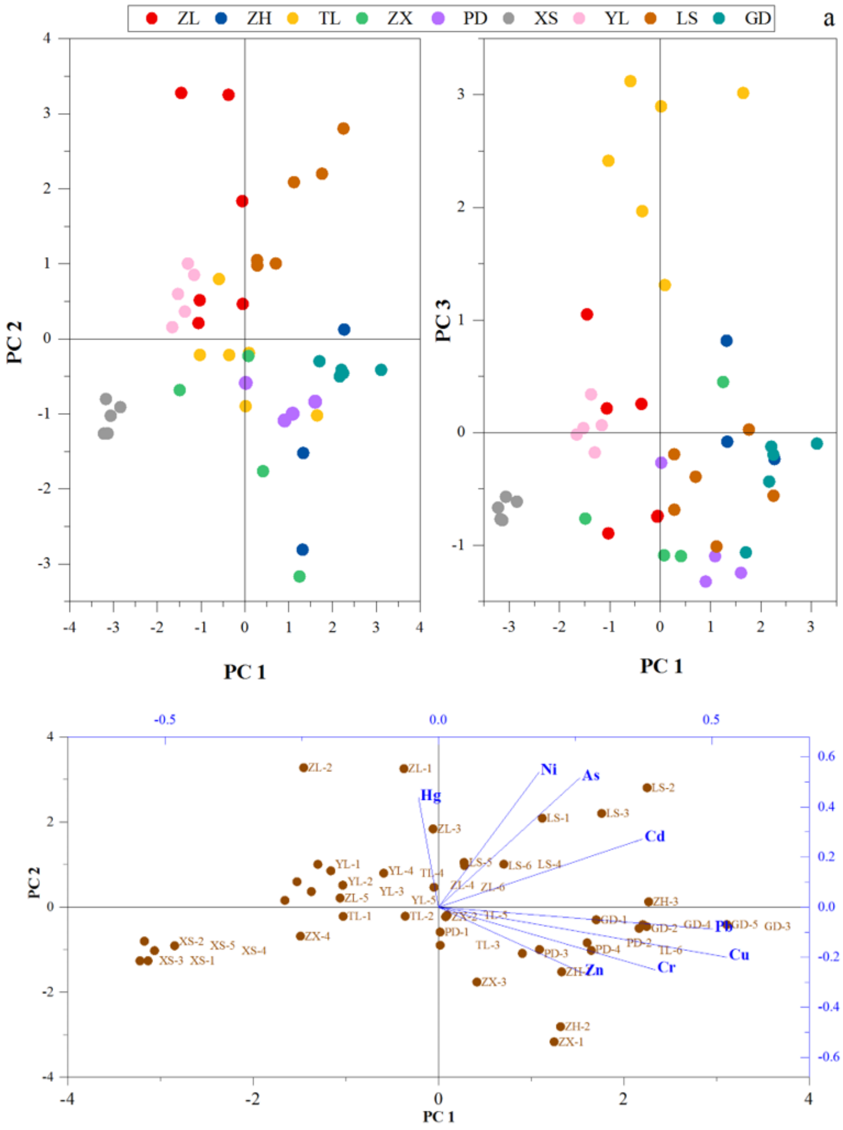
ZL ZH TL ZX PD XS YL LS GD
a
PC 1
PC 2
PC 3
Ni
As
Hg
Cd
Pb
Cu
Cr
Zn
ZL-1
ZL-2
ZL-3
ZL-5
LS-1
LS-2
LS-3
LS-5
LS-6
YL-1
YL-2
YL-3
YL-4
XS-1
XS-2
XS-3
XS-4
XS-5
ZX-1
ZX-3
ZX-4
ZH-2
ZH-3
TL-1
TL-2
TL-3
PD-1
PD-3
PD-4
GD-1
GD-2
GD-3
GD-5

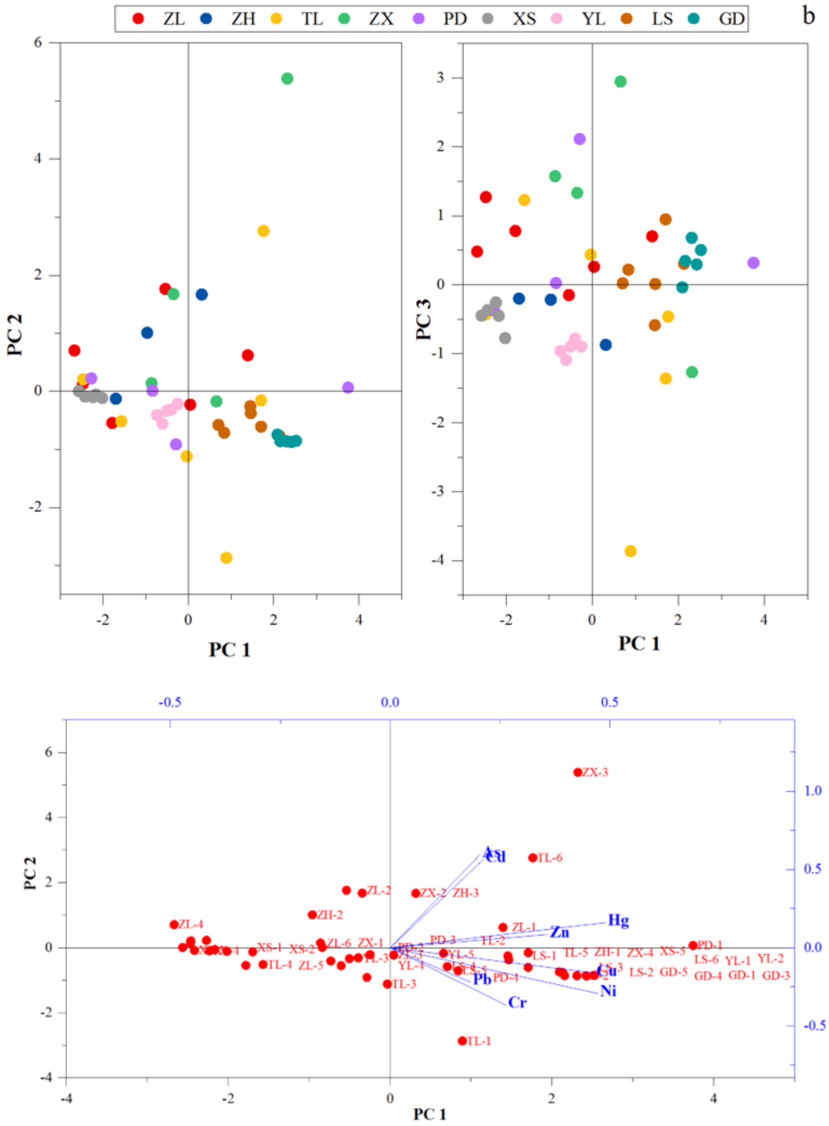
ZL ZH TL ZX PD XS YL LS GD
b
PC 1
PC 2
PC 3
Hg
Zn
Cd
As
Cu
Ni
Cr
Pb

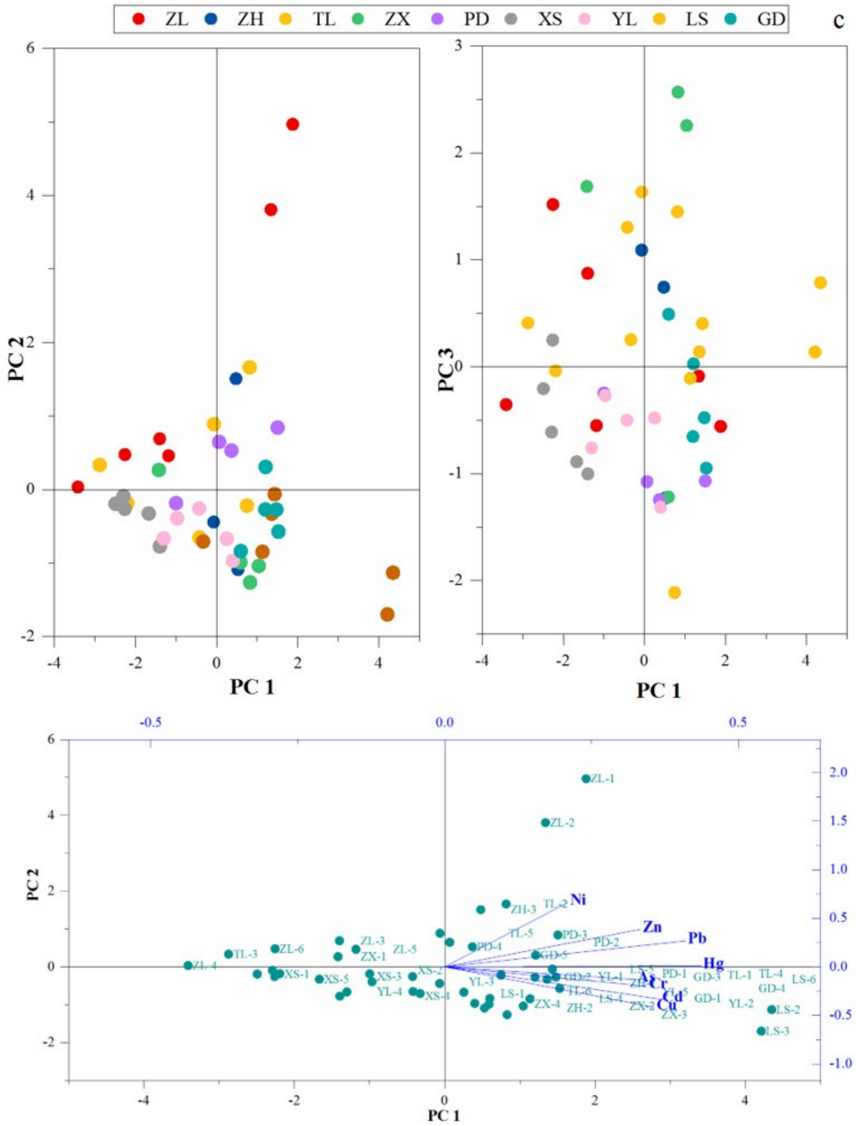

Figure S4. Principal component analysis (PCA) of eight target elements in samples collected from nine study areas. a) PCA of all determined soils with loading plot and score plot (n=176); b) PCA of all determined vegetable edible parts with loading plot and score plot (n=176); and c) PCA of all determined vegetable leaves with loading plot and score plot (n=176). Refer the denotes to Figure S1.

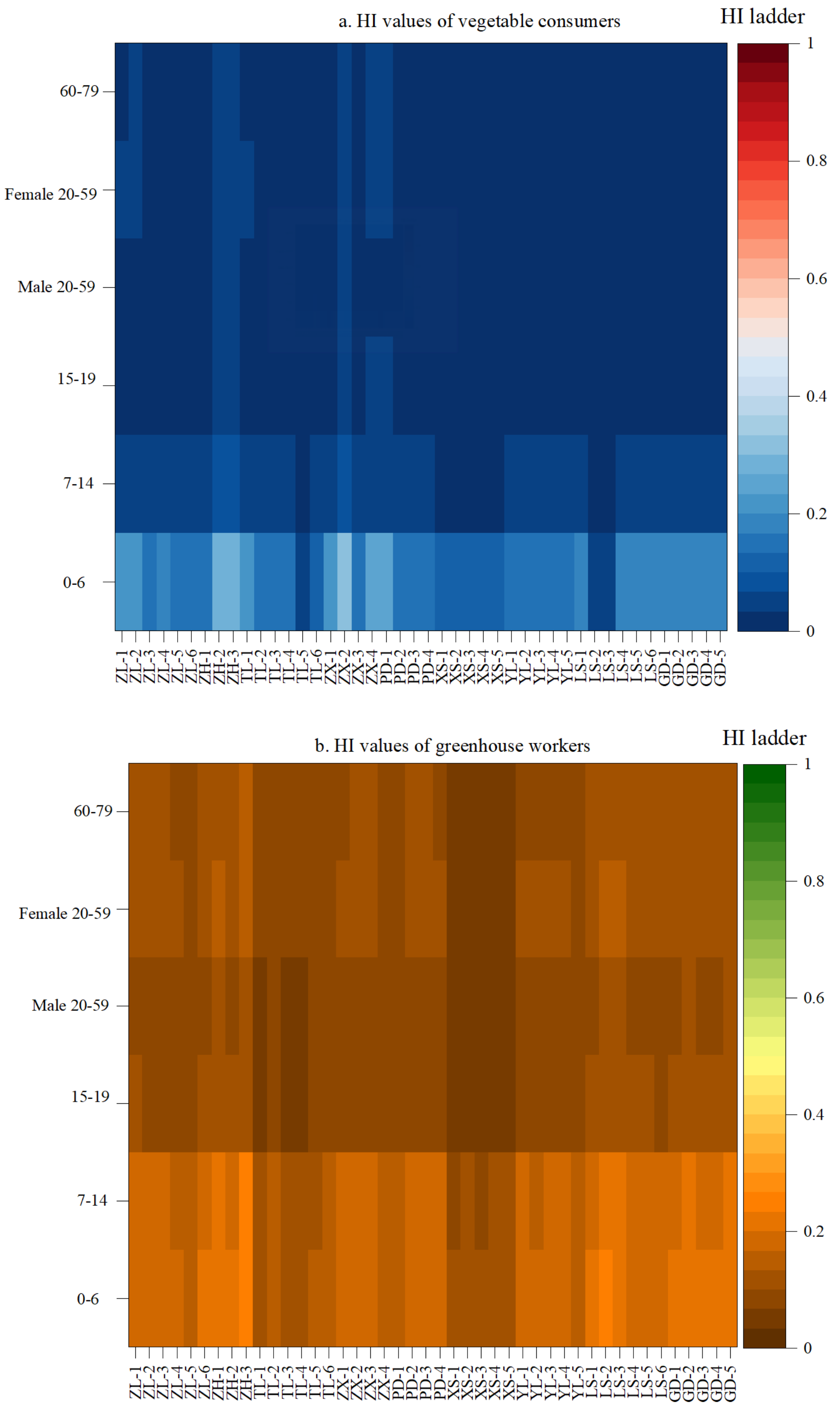

Figure S5. Heatmap of non-carcinogenic risks based on HI values from collected samples in nine study areas. a) HI values of vegetable consumers, b) HI values of greenhouse workers. Refer the denotes to Figure S1.

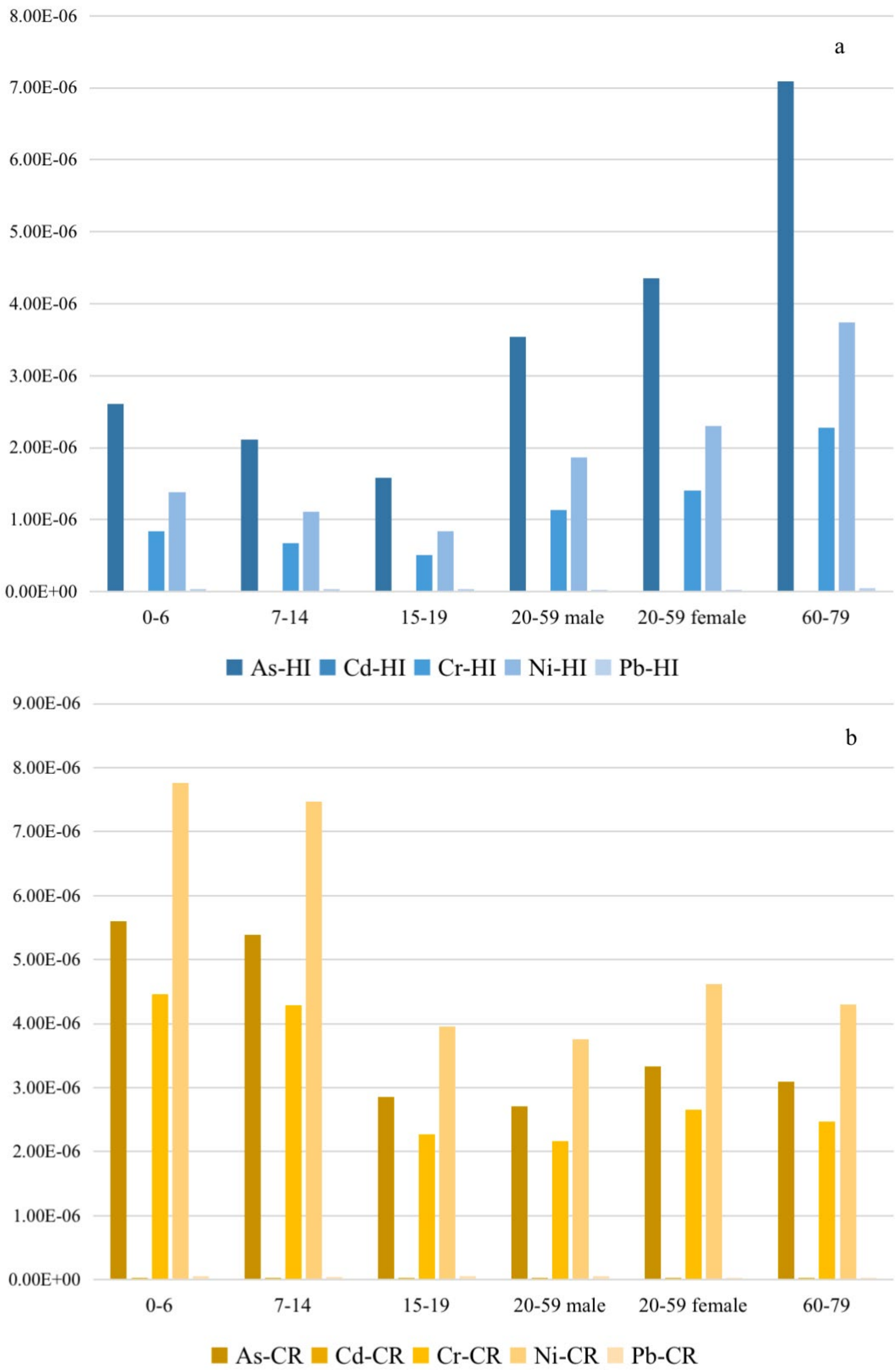

Figure S6. Contributions of different elements to carcinogenic risks of people in different age groups. a) Vegetable consumers, b) greenhouse workers. Refer the denotes to Figure S1.

Table S1. Physical-chemical characteristic of soils and overview of production information in nine study areas.

Area	Location / abbreviation	pH	EC (μS/cm)	Clay (%)	Silt (%)	Sand (%)	Organic matter (%)	Planting time (a)	Annual productive time (d)	Planting area (ha)	Fertilizer type	Pesticide number
1	Bajiao Village, Zilingpu Town, Dongbao District/Zilingpu (ZL)	6.37 ± 0.44	963 ± 96	22	15	63	2.42 ± 0.17	9	270	30	In- and organic	3 ~5
2	Tandian Village, Zhanghe New District/Zhanghe (ZH)	7.18 ± 0.35	851 ± 77	25	14	61	2.88 ± 0.11	13	270	400	In- and organic	3 ~5
3	Tuanlin Town, Duodao District/Tuanlin (TL)	7.57 ± 0.33	721 ± 32	25	16	59	3.68 ± 0.11	15	210	120	In- and organic	4 ~ 6
4	Phosphate Rock Town, Zhongxiang City/Zhongxiang (ZX)	6.48 ± 0.31	961 ± 21	23	15	62	2.96 ± 0.15	9	250	80	Inorganic	3 ~5
5	Pengdun village, Shipai Town, Zhongxiang City/Pengdun (PD)	6.72 ± 0.49	987 ± 62	26	11	63	2.67 ± 0.21	10	240	266.67	Organic	Over 6
6	Xinshi Town, Jingshan City/Xinshi (XS)	6.11 ± 0.53	894 ± 77	25	13	62	3.33 ± 0.27	6	220	26.67	In- and organic	3 ~5
7	Yonglong Town, Jingshan City/Yonglong (YL)	6.56 ± 0.38	923 ± 58	25	11	64	3.39 ± 0.16	9	270	46.67	In- and organic	3 ~5
8	Guangmang Village, Lishi Town, Shayang County/Lishi (LS)	7.23 ± 0.45	866 ± 72	24	14	62	2.95 ± 0.24	11	260	133.33	Organic	Over 6

9	Guandang Town, Shayang County/Guandang (GD)	7.31 ± 0.36	787 ± 66	25	14	61	3.02 ± 0.19	14	270	43.87	Organic	Over 6

Table S2. Classification of baseline values for Igeo, PI, NIPI, E_r and RI

Value [33]	Degree of pollution	Value [39]	Degree of pollution	Value [37]	Degree of pollution	Value [38]	Degree of risk	Value [38]	Degree of risk
Igeo ≤ 0	Unpolluted	PI ≤ 1	Unpolluted	NIPI ≤ 0.7	Unpolluted	E_r < 40	Low	RI < 150	Low
0 < Igeo ≤ 1	Unpolluted to moderately polluted	1 < PI ≤ 2	Slightly polluted	0.7 < NIPI ≤ 1	Waring threshold	40 ≤ E_r < 80	Moderate	150 ≤ RI < 300	Moderate
1 < Igeo ≤ 2	Moderately polluted	2 < PI ≤3	Mildly polluted	1 < NIPI ≤ 2	Low	80 ≤ E_r < 160	Considerable	300 ≤ RI < 600	Considerable
2 < Igeo ≤ 3	Moderately to strongly polluted	3 < PI ≤ 5	Moderately polluted	2 < NIPI ≤ 3	Moderate	160 ≤ E_r < 320	High	600 ≤ RI < 1200	High
3 < Igeo ≤ 4	Strongly polluted	PI > 5	Highly polluted	NIPI > 3	Severe	E_r ≥ 320	Disastrous	RI ≥ 1200	Disastrous
4 < Igeo ≤ 5	Strongly to extremely polluted								
Igeo > 5	Extremely polluted								

Table S3. Parameters for health risk assessment

Parameters		Abbreviation	Child	Adolescent		Adult		Old	Unit
Age [40]			0-6 [41]	7-14	15-19	Male 20-59 [41]	Female 20-59 [41]	60-79 [41]	
Body weight [40]		BW	22.5	36.4	68.75	72.48	58.85	63.29	kg
Daily vegetable intake [40]	Leaf vegetable	$IR_{vegetable}$	0.037	0.019					kg DW/kg BW per day
	Other vegetable		0.125	0.054					
Exposure frequency [40]		$EF_{vegetable}$	365						d/a
		EF_{soil}	1/3 of adults	Working days per year (Table S1)					
Exposure duration [40]		$ED_{vegetable}$	4	12	17	40		70	year
		ED_{soil}	Production duration of greenhouse						
Conversion factor [45]		CF	10^{-6}						kg/mg
Average time [40]		AT	365×ED for HQ, lifetime (28835 d) for CR						d
Soil ingestion rate [43]		$IR_{soil-ingestion}$	200	100					mg/d
Soil inhale rate [1]		$IR_{soil-inhale}$	7.6	20					m^3/d
Exposure time		ET	1	6					h
Lifetime [40]		LT	79						year
Skin surface area exposed for daily contact [2]		SA	2800	5700					cm^2/d
Soil adherence factor of skin [40]		ADF	0.2	0.07					mg/cm^2
Dermal absorption factor from the soil [40]		ABS	0.001	0.1					unitless
Particle emission factor [40]		PEF	1.36×10^9						m^3/kg

Continued Table S3

Parameters	Abbreviation	Unit	As	Cd	Cr	Cu	Hg	Ni	Pb	Zn
Reference dose [43]	RfD	(mg/kg)/d	0.0003	0.0001	0.003	0.04	0.001	0.02	0.0035	0.3
Reference concentration [43]	RfC	mg/m^3	0.000015	0.00001	0.00006	/	0.0003	0.00009	/	/
Inhalation unit risk [43]	IUR	μg/m^{-3}	0.0043	0.0018	0.012	/	/	0.00026	0.000012	/
Oral slope factor of the carcinogen [43]	SFO	(mg/kg)/d	1.5	/	0.42	/	/	0.84	0.0085	/

[1] Integrated Risk Information System (IRIS). Washington, DC, USA. Available at http://www.epa.gov/ncea/iris/index.html (USEPA, 2007).

[2] A-Z Index of Substances. Washington, DC, USA. Available at http://www2.epa.gov/home/az-index (USEPA, 2008).

Table S4. The Nemerow integrated pollution index and risk level assessment results of target elements in greenhouse of the nine study areas of Jingmen City

**Study area**	**NIPI** [34]			**Risk level**	**NIPI'** [35]			**Risk level**
	Mean	Min	Max		Mean	Min	Max	
Zilingpu	3.39	0	13.47	Severe	2.17	0	12.19	*Moderate*
Zhanghe	3.70	0	17.88	Severe	2.59	0	16.18	*Moderate*
Tuanlin	3.12	0	13.38	Severe	2.06	0	12.11	*Moderate*
Zhongxiang	3.29	0	14.84	Severe	1.76	0	9.82	*Low*
Pengdun	2.83	0	9.90	Moderate	1.39	0	6.55	*Low*
Xinshi	2.24	0	8.39	Moderate	1.18	0	6.5	*Low*
Yonglong	2.74	0	10.13	Moderate	1.58	0	8.71	*Low*
Lishi	3.69	0	15.40	Severe	2.79	0	17.66	*Moderate*
Guanndang	3.40	0	14.19	Severe	2.61	0	16.27	*Moderate*

[1] Italic risk levels indicate downgraded risk level compared with that based on the old geochemical background values.

Table S5a. Er values of target elements (As, Cd, Cr, Cu) in greenhouse of the nine study areas in Jingmen City [1]

**Area**	**No.**	**Sample number**	**As**			**Cd**			**Cr**			**Cu**		
			Er [34]	Er′ [35]	Level	Er [34]	Er′ [35]	Level	Er [34]	Er′ [35]	Level	Er [34]	Er′ [35]	Level
ZL n=24	1	ZL-1	12.24	12.05	Low	441.51	399.68	Disastrous	0.42	0.46	Low	0.02	0.03	Low
	2	ZL-2	12.96	12.76	Low	360.18	326.05	Disastrous	0.26	0.29	Low	0.02	0.03	Low
	3	ZL-3	11.32	11.14	Low	437.63	396.17	Disastrous	0.28	0.31	Low	0.02	0.03	Low
	4	ZL-4	10.93	10.75	Low	271.10	245.42	High	0.76	0.83	Low	0.03	0.03	Low
	5	ZL-5	9.13	8.99	Low	364.05	329.56	Disastrous	0.55	0.61	Low	0.03	0.03	Low
	6	ZL-6	11.38	11.20	Low	302.08	273.47	High	1.50	1.65	Low	0.03	0.03	Low
ZH n=15	7	ZH-1	10.92	10.75	Low	441.51	399.68	Disastrous	1.75	1.93	Low	0.03	0.03	Low
	8	ZH-2	9.68	9.53	Low	367.92	333.07	Disastrous	1.54	1.70	Low	0.03	0.04	Low
	9	ZH-3	10.35	10.18	Low	596.42	539.92	Disastrous	1.89	2.08	Low	0.03	0.04	Low
TL n=24	10	TL-1	11.07	10.89	Low	302.08	273.47	High	0.39	0.43	Low	0.03	0.03	Low
	11	TL-2	11.12	10.94	Low	468.62	424.22	Disastrous	0.27	0.29	Low	0.03	0.03	Low
	12	TL-3	11.34	11.16	Low	263.35	238.41	High	0.30	0.33	Low	0.03	0.04	Low
	13	TL-4	11.53	11.34	Low	274.97	248.92	High	0.30	0.33	Low	0.03	0.03	Low
	14	TL-5	12.52	12.31	Low	309.83	280.48	High	0.24	0.26	Low	0.03	0.03	Low
	15	TL-6	12.47	12.27	Low	302.08	273.47	High	0.27	0.29	Low	0.04	0.04	Low
ZX n=20	16	ZX-1	9.06	9.21	Low	305.96	202.40	High	1.54	1.63	Low	0.03	0.03	Low
	17	ZX-2	10.16	10.33	Low	515.09	340.75	Disastrous	0.85	0.90	Low	0.03	0.03	Low
	18	ZX-3	9.83	10.00	Low	298.21	197.28	High	1.71	1.82	Low	0.03	0.03	Low
	19	ZX-4	8.20	8.34	Low	329.19	217.77	**Disastrous-High**	0.77	0.82	Low	0.03	0.03	Low
PD n=20	20	PD-1	10.27	10.44	Low	205.26	135.79	High	0.65	0.69	Low	0.03	0.03	Low
	21	PD-2	10.74	10.92	Low	302.08	199.84	High	1.86	1.98	Low	0.03	0.03	Low
	22	PD-3	10.74	10.92	Low	251.74	166.53	High	1.93	2.05	Low	0.03	0.03	Low
	23	PD-4	10.05	10.22	Low	321.45	212.65	**Disastrous-High**	1.51	1.60	Low	0.03	0.03	Low
XS n=20	24	XS-1	8.36	8.36	Low	206.62	177.69	High	0.31	0.34	Low	0.02	0.02	Low
	25	XS-2	8.81	8.81	Low	224.04	192.68	High	0.29	0.31	Low	0.02	0.02	Low

	26	XS-3	8.42	8.42	Low	194.59	167.35	High	0.29	0.31	Low	0.02	0.03	Low
	27	XS-4	8.92	8.92	Low	227.12	195.32	High	0.36	0.39	Low	0.02	0.02	Low
	28	XS-5	8.97	8.97	Low	235.79	202.78	High	0.34	0.37	Low	0.02	0.03	Low
YL n=20	29	YL-1	11.57	11.57	Low	289.35	248.84	High	0.60	0.65	Low	0.03	0.03	Low
	30	YL-2	11.02	11.02	Low	292.14	251.24	High	0.52	0.57	Low	0.02	0.03	Low
	31	YL-3	11.10	11.10	Low	309.85	266.47	High	0.60	0.66	Low	0.03	0.03	Low
	32	YL-4	11.40	11.40	Low	304.56	261.92	High	0.61	0.67	Low	0.02	0.03	Low
	33	YL-5	10.04	10.04	Low	293.98	252.82	High	0.53	0.58	Low	0.03	0.03	Low
LS n=24	34	LS-1	12.71	12.92	Low	372.53	427.17	Disastrous	0.85	0.96	Low	0.03	0.04	Low
	35	LS-2	14.38	14.62	Low	509.48	584.21	Disastrous	0.92	1.04	Low	0.03	0.04	Low
	36	LS-3	14.09	14.32	Low	501.37	574.90	Disastrous	0.92	1.04	Low	0.03	0.04	Low
	37	LS-4	12.64	12.85	Low	381.18	437.09	Disastrous	0.79	0.89	Low	0.03	0.04	Low
	38	LS-5	13.26	13.48	Low	342.82	393.10	Disastrous	0.82	0.93	Low	0.03	0.04	Low
	39	LS-6	11.65	11.84	Low	348.22	399.30	Disastrous	0.82	0.93	Low	0.03	0.04	Low
GD n=20	40	GD-1	10.07	10.24	Low	403.82	463.05	Disastrous	1.29	1.46	Low	0.03	0.04	Low
	41	GD-2	10.78	10.96	Low	447.99	513.70	Disastrous	1.22	1.38	Low	0.03	0.04	Low
	42	GD-3	10.56	10.74	Low	376.36	431.56	Disastrous	1.23	1.39	Low	0.03	0.04	Low
	43	GD-4	10.80	10.98	Low	370.71	425.08	Disastrous	1.24	1.40	Low	0.03	0.04	Low
	44	GD-5	10.83	11.01	Low	412.52	473.02	Disastrous	1.26	1.43	Low	0.04	0.04	Low
Average value			10.87	10.89	Low	342.67	318.05	**Disastrous-High**	0.85	0.93	Low	0.03	0.03	Low

Table S5b. Er values of target elements (Hg, Ni, Pb, Zn) in greenhouse of the nine study areas in Jingmen City [1]

**Area**	**No.**	**Sample number**	**Hg**			**Ni**			**Pb**			**Zn**		
			Er [34]	Er′ [35]	Level	Er [34]	Er′ [35]	Level	Er [34]	Er′ [35]	Level	Er [34]	Er′ [35]	Level
ZL	1	ZL-1	43.72	61.25	Moderate	51.82	5.79	**Moderate-Low**	3.85	3.32	Low	0.00	0.00	Low

n=24	2	ZL-2	48.57	68.05	Moderate	47.79	5.34	**Moderate-Low**	3.29	2.83	Low	0.00	0.00	Low
	3	ZL-3	25.03	35.07	Low	49.95	5.58	**Moderate-Low**	4.90	4.22	Low	0.00	0.00	Low
	4	ZL-4	14.08	19.73	Low	50.67	5.66	**Moderate-Low**	3.22	2.77	Low	0.00	0.00	Low
	5	ZL-5	35.02	49.07	**Low- Moderate**	42.03	4.69	**Moderate-Low**	3.64	3.13	Low	0.00	0.00	Low
	6	ZL-6	29.36	41.13	**Low- Moderate**	44.77	5.00	**Moderate-Low**	4.13	3.56	Low	0.00	0.00	Low
ZH	7	ZH-1	13.16	18.44	Low	37.57	4.20	Low	4.13	3.56	Low	0.00	0.00	Low
n=15	8	ZH-2	10.33	14.47	Low	36.28	4.05	Low	4.13	3.56	Low	0.00	0.00	Low
	9	ZH-3	29.71	41.62	**Low- Moderate**	43.76	4.89	**Moderate-Low**	4.20	3.62	Low	0.00	0.00	Low
	10	TL-1	29.36	41.14	**Low- Moderate**	40.31	4.50	**Moderate-Low**	2.94	2.53	Low	0.00	0.00	Low
	11	TL-2	19.43	27.22	Low	39.15	4.37	Low	3.15	2.71	Low	0.00	0.00	Low
TL	12	TL-3	23.28	32.62	Low	41.17	4.60	**Moderate-Low**	3.43	2.95	Low	0.00	0.00	Low
n=24	13	TL-4	46.59	65.27	Moderate	42.32	4.73	**Moderate-Low**	3.64	3.13	Low	0.00	0.00	Low
	14	TL-5	11.40	15.97	Low	43.33	4.84	**Moderate-Low**	4.13	3.56	Low	0.00	0.00	Low
	15	TL-6	21.35	29.91	Low	39.15	4.37	Low	4.69	4.04	Low	0.00	0.00	Low
	16	ZX-1	15.54	24.14	Low	33.68	3.35	Low	4.97	4.82	Low	0.00	0.00	Low
ZX	17	ZX-2	8.24	12.79	Low	43.19	4.30	**Moderate-Low**	3.99	3.87	Low	0.00	0.00	Low
n=20	18	ZX-3	14.28	22.19	Low	39.44	3.92	Low	4.55	4.42	Low	0.00	0.00	Low
	19	ZX-4	21.46	33.34	Low	43.47	4.32	**Moderate-Low**	3.57	3.47	Low	0.00	0.00	Low
	20	PD-1	19.32	30.02	Low	46.35	4.61	**Moderate-Low**	4.90	4.76	Low	0.00	0.00	Low
PD	21	PD-2	15.87	24.66	Low	47.22	4.70	**Moderate-Low**	4.83	4.69	Low	0.00	0.00	Low
n=20	22	PD-3	14.82	23.03	Low	45.49	4.52	**Moderate-Low**	4.55	4.42	Low	0.00	0.00	Low
	23	PD-4	12.31	19.12	Low	47.36	4.71	**Moderate-Low**	4.13	4.01	Low	0.00	0.00	Low
	24	XS-1	11.02	13.99	Low	41.00	4.43	**Moderate-Low**	3.23	2.89	Low	0.00	0.00	Low
XS	25	XS-2	11.77	14.95	Low	42.29	4.57	**Moderate-Low**	3.07	2.74	Low	0.00	0.00	Low
n=20	26	XS-3	10.49	13.32	Low	41.53	4.49	**Moderate-Low**	2.88	2.58	Low	0.00	0.00	Low
	27	XS-4	11.01	13.98	Low	41.13	4.45	**Moderate-Low**	2.80	2.50	Low	0.00	0.00	Low
	28	XS-5	11.42	14.50	Low	41.86	4.53	**Moderate-Low**	2.97	2.65	Low	0.00	0.00	Low
YL	29	YL-1	26.18	33.24	Low	45.51	4.92	**Moderate-Low**	3.49	3.12	Low	0.00	0.00	Low

n=20	30	YL-2	25.50	32.38	Low	43.73	4.73	**Moderate-Low**	3.68	3.29	Low	0.00	0.00	Low
	31	YL-3	26.24	33.32	Low	41.64	4.50	**Moderate-Low**	3.49	3.12	Low	0.00	0.00	Low
	32	YL-4	27.58	35.02	Low	44.48	4.81	**Moderate-Low**	3.72	3.32	Low	0.00	0.00	Low
	33	YL-5	26.08	33.11	Low	42.92	4.64	**Moderate-Low**	3.43	3.06	Low	0.00	0.00	Low
	34	LS-1	19.07	23.99	Low	57.66	7.01	**Moderate-Low**	4.18	3.78	Low	0.00	0.00	Low
	35	LS-2	19.81	24.92	Low	56.22	6.83	**Moderate-Low**	4.41	3.99	Low	0.00	0.00	Low
LS	36	LS-3	21.24	26.72	Low	51.33	6.24	**Moderate-Low**	3.84	3.48	Low	0.00	0.00	Low
n=24	37	LS-4	16.58	20.85	Low	48.51	5.89	**Moderate-Low**	4.05	3.67	Low	0.00	0.00	Low
	38	LS-5	16.64	20.93	Low	47.14	5.73	**Moderate-Low**	3.87	3.50	Low	0.00	0.00	Low
	39	LS-6	18.24	22.95	Low	51.16	6.22	**Moderate-Low**	3.95	3.57	Low	0.00	0.00	Low
	40	GD-1	19.16	24.10	Low	47.82	5.81	**Moderate-Low**	4.80	4.34	Low	0.00	0.00	Low
GD	41	GD-2	16.31	20.51	Low	45.17	5.49	**Moderate-Low**	4.99	4.52	Low	0.00	0.00	Low
n=20	42	GD-3	20.31	25.55	Low	48.06	5.84	**Moderate-Low**	5.27	4.77	Low	0.00	0.00	Low
	43	GD-4	19.08	24.00	Low	48.50	5.89	**Moderate-Low**	5.17	4.68	Low	0.00	0.00	Low
	44	GD-5	18.33	23.06	Low	49.82	6.05	**Moderate-Low**	5.55	5.02	Low	0.00	0.00	Low
	Average value		20.78	28.31	Low	44.86	5.00	**Moderate-Low**	4.00	3.60	Low	0.00	0.00	Low

[1] ZL-1, sample No. 1, is the first sampled greenhouse in Zilingpu; ZH-1, sample No. 7, is the first sampled greenhouse in Zhanghe; TL-1, sample No. 10, is the first sampled greenhouse in Tuanlin; ZX-1, sample No. 16, is the first sampled greenhouse in Zhongxiang; PD-1, sample No. 20, is the first sampled greenhouse in Pengdun; XS-1, sample No. 24, is the first sampled greenhouse in Xinshi; YL-1, sample No. 29, is the first sampled greenhouse in Yonglong; LS-1, sample No. 34, is the first sampled greenhouse in Lishi; and GD-1, sample No. 40, is the first sampled greenhouse in Guandang.

Table S6. RI values and risk level assessment results of target elements in greenhouse of the nine study areas of Jingmen City [1]

**Study area**	**RI** [34]			**Risk level**	**RI′** [35]			**Risk level**
	Mean	Min	Max		Mean	Min	Max	
Zilingpu	459.05	350.79	553.58	Considerable risk	394.63	285.20	482.56	*Moderate to considerable risk*
Zhanghe	541.78	429.91	686.36	Considerable to high risk	469.11	366.41	602.35	Considerable to high risk
Tuanlin	401.96	342.91	541.76	Considerable risk	344.75	290.10	469.79	*Moderate to considerable risk*
Zhongxiang	431.77	368.06	581.54	Considerable risk	281.57	239.65	372.97	*Moderate to considerable risk*
Pengdun	348.89	286.79	396.84	Moderate to considerable risk	224.25	186.33	252.35	*Moderate risk*
Xinshi	282.36	258.22	301.38	Moderate to considerable risk	217.54	196.49	233.83	*Moderate risk*
Yonglong	383.13	376.63	392.95	Considerable risk	309.25	302.37	319.20	Considerable risk
Lishi	497.93	424.58	605.25	Considerable to high risk	517.02	437.70	635.64	Considerable to high risk
Guanndang	485.84	455.54	526.50	Considerable risk	507.45	366.82	556.60	Considerable risk

[1] Italic risk levels indicate downgraded risk level compared with that based on the old geochemical background values.

Table S7. Concentrations of target elements in vegetable edible parts of greenhouses in Jingmen City (mean ± SD) (dry weight/DW) [1]

No.	Area		Vegetable type	As (mg/kg)	Cd (mg/kg)	Cr (mg/kg)	Cu (μg/kg)	Hg (μg/kg)	Ni (mg/kg)	Pb (mg/kg)	Zn (mg/kg)
1	ZL n=24	1	Green eggplant	***5.84 ± 0.07***	***1.19 ± 0.36***	***6.13 ± 0.73***	36.37 ± 1.70	***16.33 ± 1.65***	***6.49 ± 2.15***	***8.67 ± 0.05***	36.84 ± 1.69
		2	Yellow cucumber	***6.63 ± 0.24***	***1.47 ± 0.09***	***3.84 ± 0.28***	30.28 ± 2.64	***11.01 ± 0.33***	***5.24 ± 2.06***	***6.43 ± 0.76***	28.85 ± 0.10
		3	Cucumber	***6.13 ± 0.13***	***0.47 ± 0.03***	***6.02 ± 0.68***	41.33 ± 2.75	***11.50 ± 0.45***	***5.11 ± 2.41***	***7.77 ± 0.26***	44.33 ± 1.10
		4	Tomato	***5.69 ± 0.18***	***0.85 ± 0.36***	***3.28 ± 0.16***	36.77 ± 4.43	6.48 ± 0.23	***3.14 ± 1.75***	***6.43 ± 0.55***	15.62 ± 0.07
		5	Green chili	***3.69 ± 0.14***	***0.81 ± 0.44***	***3.43 ± 0.19***	41.73 ± 2.20	5.13 ± 0.47	***5.80 ± 2.74***	***7.47 ± 0.30***	19.34 ± 0.55
		6	Small cushaw	***4.88 ± 0.17***	***0.82 ± 0.25***	***3.32 ± 0.25***	35.56 ± 3.56	5.06 ± 0.22	***3.48 ± 1.56***	***8.07 ± 0.42***	21.27 ± 0.10
2	ZH n=15	7	Long purple eggplant	***5.17 ± 0.17***	***0.77 ± 0.38***	***5.92 ± 0.69***	55.81 ± 4.28	6.21 ± 0.70	***4.64 ± 2.88***	***5.68 ± 0.85***	16.60 ± 0.64
		8	Green chili	***12.64 ± 0.43***	***0.63 ± 0.20***	***5.61 ± 0.16***	44.38 ± 4.75	7.70 ± 0.47	***5.71 ± 5.04***	***6.13 ± 0.55***	16.90 ± 0.11
		9	Cauliflower	***11.62 ± 0.23***	***0.78 ± 0.25***	***6.54 ± 1.00***	33.98 ± 3.57	***10.75 ± 0.35***	***4.64 ± 3.13***	***6.43 ± 0.92***	54.60 ± 0.13
		10	Cherry tomato	***5.80 ± 0.17***	***0.70 ± 0.37***	***27.56 ± 0.47***	44.27 ± 2.95	6.20 ± 0.48	***8.42 ± 4.29***	***5.68 ± 0.12***	21.49 ± 0.11
3	TL n=24	11	Long purple eggplant	***4.79 ± 0.06***	***0.65 ± 0.25***	***4.77 ± 0.70***	32.60 ± 2.63	5.80 ± 0.21	***3.52 ± 1.56***	***5.83 ± 0.19***	28.99 ± 0.20
		12	Pumpkin	***4.98 ± 0.11***	***0.68 ± 0.20***	***5.57 ± 0.53***	44.56 ± 1.70	5.22 ± 0.36	***7.86 ± 5.26***	***8.37 ± 0.15***	40.67 ± 0.15
		13	Cucumber	***4.76 ± 0.14***	***0.60 ± 0.13***	***5.09 ± 0.36***	45.59 ± 3.74	5.74 ± 0.53	***3.87 ± 2.57***	***8.52 ± 0.85***	31.49 ± 0.04
		14	Lettuce	***8.49 ± 0.14***	***0.52 ± 0.06***	***6.95 ± 0.84***	60.07 ± 5.28	***11.59 ± 0.41***	***8.29 ± 3.53***	***5.53 ± 0.99***	50.53 ± 0.26
		15	Garland chrysanthemum	***17.52 ± 0.05***	***0.81 ± 0.11***	***5.61 ± 0.51***	66.56 ± 3.45	***14.87 ± 0.38***	***5.15 ± 3.67***	***5.68 ± 0.52***	41.03 ± 0.16
4	ZX n=20	16	Cauliflower	***6.77 ± 0.40***	***0.87 ± 0.20***	***5.61 ± 0.32***	35.04 ± 1.95	7.21 ± 0.20	***4.30 ± 1.97***	***9.72 ± 0.70***	35.34 ± 0.19
		17	Asparagus lettuce	***13.41 ± 0.16***	***1.00 ± 0.40***	***4.15 ± 0.06***	50.15 ± 6.01	9.59 ± 0.31	***5.15 ± 2.07***	***8.07 ± 0.28***	14.18 ± 0.05
		18	Chinese cabbage	***14.96 ± 0.39***	***2.88 ± 0.16***	***7.07 ± 0.68***	40.52 ± 4.51	***12.21 ± 0.64***	***5.50 ± 3.78***	***5.83 ± 0.14***	53.18 ± 0.10
		19	Green chili	***8.66 ± 0.25***	***1.03 ± 0.32***	***6.13 ± 0.57***	74.46 ± 2.07	8.14 ± 0.28	***5.54 ± 1.87***	***10.91 ± 0.61***	21.26 ± 0.12
5	PD n=20	20	Cherry tomato	***8.51 ± 0.36***	***0.99 ± 0.07***	***8.53 ± 0.69***	99.39 ± 3.40	***15.54 ± 1.02***	***7.52 ± 4.60***	***7.47 ± 0.26***	49.77 ± 0.61
		21	Mini-cucumber	***5.61 ± 0.12***	***0.65 ± 0.09***	***5.51 ± 0.32***	45.70 ± 2.76	7.30 ± 0.15	***4.34 ± 3.17***	***7.03 ± 0.37***	44.12 ± 0.10
		22	Purple eggplant	***4.95 ± 0.15***	***0.85 ± 0.41***	***5.61 ± 0.67***	21.37 ± 4.28	7.30 ± 0.56	***4.81 ± 4.12***	***6.28 ± 0.90***	16.86 ± 0.26
		23	Green chili	***5.30 ± 0.20***	***0.60 ± 0.15***	***6.13 ± 0.37***	55.31 ± 4.14	8.24 ± 0.15	***4.73 ± 3.40***	***10.16 ± 0.92***	32.10 ± 0.88
6	XS n=20	24	Cucumber	***3.59 ± 0.12***	***0.59 ± 0.26***	***3.94 ± 0.35***	23.97 ± 3.12	6.85 ± 0.28	***3.94 ± 2.98***	***5.88 ± 0.32***	28.70 ± 0.38
		25	Tomato	***3.95 ± 0.19***	***0.52 ± 0.23***	***4.22 ± 0.42***	28.17 ± 3.55	7.52 ± 0.26	***4.05 ± 2.35***	***5.96 ± 0.97***	32.53 ± 0.3
		26	Bitter gourd	***4.01 ± 0.14***	***0.51 ± 0.24***	***3.96 ± 0.51***	25.68 ± 3.33	6.32 ± 0.24	***3.99 ± 3.61***	***6.14 ± 0.85***	33.05 ± 0.29

7	YL<br>n=20	27	Purple eggplant	***3.36 ± 0.10***	***0.63 ± 0.20***	***3.67 ± 0.39***	33.12 ± 2.96	6.65 ± 0.25	***4.22 ± 2.55***	***5.98 ± 0.65***	29.62 ± 0.35
		28	Green chili	***3.55 ± 0.12***	***0.58 ± 0.28***	***5.62 ± 0.41***	26.69 ± 4.10	7.62 ± 0.22	***3.98 ± 2.76***	***5.86 ± 0.56***	35.62 ± 0.30
		29	Tomato	***4.53 ± 0.15***	***0.69 ± 0.25***	***7.62 ± 0.38***	31.86 ± 3.29	8.17 ± 0.29	***5.95 ± 3.25***	***6.33 ± 0.35***	38.45 ± 0.22
		30	Green chili	***4.44 ± 0.11***	***0.71 ± 0.20***	***8.62 ± 0.42***	33.82 ± 3.00	9.64 ± 0.31	***5.32 ± 3.55***	***6.52 ± 0.62***	39.66 ± 0.25
		31	Long purple eggplant	***4.32 ± 0.16***	***0.78 ± 0.19***	***8.67 ± 0.37***	35.94 ± 2.85	9.66 ± 0.26	***5.34 ± 3.14***	***6.66 ± 0.55***	39.45 ± 0.24
		32	Cucumber	***4.15 ± 0.14***	***0.69 ± 0.33***	***8.95 ± 0.26***	36.42 ± 2.40	8.62 ± 0.25	***5.59 ± 2.65***	***6.22 ± 0.26***	38.45 ± 0.23
		33	Cowpea	***4.52 ± 0.11***	***0.74 ± 0.28***	***7.62 ± 0.29***	39.06 ± 3.25	9.68 ± 0.25	***5.61 ± 2.55***	***6.29 ± 0.55***	42.62 ± 0.26
8	LS<br>n=24	34	Cucumber	***6.32 ± 0.14***	***0.89 ± 0.24***	***7.95 ± 0.25***	60.70 ± 3.33	**11.57 ± 0.24**	***7.65 ± 2.68***	***6.62 ± 0.53***	40.22 ± 0.32
		35	Chinese tarragon	***6.85 ± 0.14***	***0.84 ± 0.22***	***9.96 ± 0.22***	65.64 ± 3.29	**12.95 ± 0.22**	***7.86 ± 2.65***	***8.37 ± 0.36***	36.15 ± 0.32
		36	Water spinach	***5.95 ± 0.16***	***0.85 ± 0.18***	***8.95 ± 0.36***	66.99 ± 2.95	**12.97 ± 0.25**	***6.32 ± 2.30***	***8.96 ± 0.62***	38.22 ± 0.32
		37	White tomato eggplant	***5.99 ± 0.13***	***0.69 ± 0.31***	***7.62 ± 0.31***	61.65 ± 2.85	9.64 ± 0.27	***6.45 ± 3.16***	***7.31 ± 0.50***	39.42 ± 0.22
		38	Green chili	***5.70 ± 0.11***	***0.65 ± 0.28***	***7.22 ± 0.33***	61.99 ± 2.95	**10.46 ± 0.26**	***6.65 ± 3.62***	***7.55 ± 0.49***	38.66 ± 0.30
		39	Tomato	***6.32 ± 0.12***	***0.75 ± 0.26***	***7.16 ± 0.40***	62.15 ± 3.28	**11.04 ± 0.23**	***6.95 ± 3.03***	***7.54 ± 0.47***	48.62 ± 0.26
9	GD<br>n=20	40	Mini-cucumber	***4.89 ± 0.16***	***0.95 ± 0.22***	***8.62 ± 0.34***	75.51 ± 2.03	**13.52 ± 0.27**	***7.88 ± 3.30***	***8.31 ± 0.35***	42.40 ± 0.33
		41	Cherry tomato	***4.51 ± 0.20***	***1.00 ± 0.24***	***8.62 ± 0.36***	72.62 ± 2.41	**12.85 ± 0.25**	***7.62 ± 2.64***	***8.65 ± 0.39***	42.37 ± 0.35
		42	Broccoli	***4.36 ± 0.16***	***0.96 ± 0.25***	***8.95 ± 0.26***	71.95 ± 2.88	**12.95 ± 0.27**	***7.51 ± 2.35***	***8.11 ± 0.42***	41.26 ± 0.22
		43	Purple ball eggplant	***5.04 ± 0.18***	***0.87 ± 0.22***	***9.32 ± 0.35***	72.57 ± 2.23	**13.85 ± 0.24**	***7.55 ± 2.57***	***8.16 ± 0.39***	43.55 ± 0.33
		44	Green chili	***5.52 ± 0.15***	***0.86 ± 0.29***	***9.67 ± 0.32***	70.97 ± 2.52	**12.95 ± 0.23**	***7.33 ± 2.62***	***7.64 ± 0.33***	41.85 ± 0.35
			Mean value	6.33	0.83	6.94	48.39	9.56	5.70	7.21	35.14
			Chinese standard [59]	0.5	Leafy 0.20<br>Others 0.05	0.50	/	10	1.0	Leafy 0.30<br>Brassica 0.20<br>Others 0.10	/
			WHO and FAO 2011 permission limits [60]	0.10	0.05	2.30	10000	10	1.50	0.50	20
			EU [61]	/	0.02-0.15	/	/	/	/	0.05-0.30	/

[1] Refer to Figure S1 and Table S6. "/" means not recommended. Bold numbers indicate exceeding the Chinese limits; underlined numbers indicate exceeding the international limits; italic bold numbers indicate exceeding both the domestic and the international limits.

Table S8. Concentrations of target elements in vegetable leaves of greenhouses in Jingmen City (mean ± SD) (dry weight/DW)

No.	Area	Vegetable type	As (mg kg-1)	Cd (mg kg-1)	Cr (mg kg-1)	Cu (μg kg-1)	Hg (μg kg-1)	Ni (mg kg-1)	Pb (mg kg-1)	Zn (mg kg-1)
1	ZL n=24	Green eggplant	823.95 ± 5.99	0.79 ± 0.09	2.66 ± 0.37	47.21 ± 1.92	78.43 ± 1.48	12.79 ± 2.01	9.92 ± 3.23	218.48 ± 1.55
		Yellow cucumber	930.66 ± 9.77	1.10 ± 0.04	3.69 ± 0.21	42.53 ± 3.35	64.44 ± 1.40	10.86 ± 2.96	8.07 ± 1.61	224.31 ± 0.14
		Cucumber	996.74 ± 9.22	0.75 ± 0.11	5.99 ± 0.32	43.74 ± 2.39	23.57 ± 0.32	6.25 ± 2.03	5.37 ± 3.20	79.55 ± 0.82
		Tomato	265.28 ± 14.29	1.02 ± 0.32	2.33 ± 0.17	21.29 ± 3.39	13.22 ± 0.32	2.38 ± 1.92	4.29 ± 2.33	96.39 ± 0.53
		Green chili	733.67 ± 12.85	1.09 ± 0.37	3.83 ± 0.17	38.94 ± 2.57	68.93 ± 0.38	5.17 ± 2.07	4.74 ± 3.74	97.76 ± 0.34
2	ZH n=15	Small cushaw	736.78 ± 12.64	0.79 ± 0.26	8.57 ± 0.31	21.87 ± 2.53	16.95 ± 0.27	5.78 ± 2.45	5.27 ± 3.75	28.64 ± 0.10
		Long purple eggplant	1581.9 ± 47.96	0.80 ± 0.29	4.98 ± 0.45	72.43 ± 4.40	34.64 ± 2.38	5.11 ± 3.60	7.77 ± 3.76	24.98 ± 0.07
		Green chili	1023.61 ± 10.2	1.40 ± 0.13	4.04 ± 1.48	94.00 ± 4.34	88.30 ± 2.10	4.81 ± 2.52	5.53 ± 1.36	70.48 ± 0.25
		Cauliflower	770.45 ± 33.23	0.80 ± 0.32	9.45 ± 0.32	31.69 ± 2.15	65.69 ± 1.21	6.09 ± 4.96	10.91 ± 4.06	51.04 ± 0.08
3	TL n=24	Cherry tomato	627.87 ± 9.69	1.09 ± 0.41	4.15 ± 0.33	85.00 ± 2.42	124.08 ± 2.62	5.45 ± 3.20	7.77 ± 0.25	32.57 ± 0.14
		Long purple eggplant	900.11 ± 5.86	0.86 ± 0.26	13.2 ± 0.26	37.08 ± 2.80	71.78 ± 2.64	9.10 ± 3.36	8.66 ± 4.93	37.77 ± 0.10
		Pumpkin	626.69 ± 30.27	0.65 ± 0.06	3.84 ± 0.25	29.07 ± 3.20	18.71 ± 0.73	4.46 ± 1.38	5.23 ± 1.36	36.29 ± 0.24
		Cucumber	719.35 ± 38.67	0.86 ± 0.14	4.46 ± 0.52	44.37 ± 4.56	30.85 ± 0.70	4.03 ± 2.76	4.33 ± 1.29	73.22 ± 0.24
		Lettuce	840.94 ± 13.56	0.52 ± 0.06	16.95 ± 0.84	60.06 ± 5.27	21.59 ± 0.40	8.29 ± 3.52	5.53 ± 2.98	100.53 ± 0.26
4	ZX n=20	Garland chrysanthemum	1715.24 ± 5.43	0.80 ± 0.10	5.60 ± 0.51	66.55 ± 3.44	34.87 ± 0.38	5.15 ± 3.66	5.68 ± 0.51	41.03 ± 0.16
		Cauliflower	1160.91 ± 15.51	1.04±0.67	4.67 ± 0.52	27.14 ± 6.57	17.14 ± 0.31	4.63 ± 2.86	7.62 ± 4.27	27.83 ± 0.10
		Asparagus lettuce	1393.8 ± 22.69	2.10 ± 0.13	11.22 ± 0.41	61.79 ± 3.80	37.01 ± 1.08	6.05 ± 4.57	6.27 ± 2.72	58.28 ± 0.32
		Chinese cabbage	1490.57 ± 38.98	2.87 ± 0.16	7.06 ± 0.68	40.51 ± 4.51	42.20 ± 0.64	5.49 ± 3.77	5.82 ± 3.13	53.18 ± 0.09
		Green chili	535.97 ± 21.64	1.73 ± 0.35	5.28 ± 0.51	96.47 ± 2.28	64.73 ± 0.33	4.33 ± 1.95	7.43 ± 3.89	79.36 ± 0.11
5	PD n=20	Cherry tomato	717.29 ± 14.24	1.04 ± 0.33	5.18 ± 0.26	54.39 ± 3.35	59.80 ± 1.92	4.81 ± 3.64	6.42 ± 0.51	33.48 ± 0.34
		Mini-cucumber	993.65 ± 20.48	0.83 ± 0.20	5.18 ± 0.29	45.88 ± 2.93	85.41 ± 2.37	3.73 ± 2.24	5.82 ± 2.24	181.28 ± 0.13
		Purple eggplant	1350.48 ± 21.98	0.70 ± 0.22	6.12 ± 0.37	73.44 ± 4.23	93.30 ± 4.38	5.28 ± 2.84	6.72 ± 2.49	198.35 ± 0.29
		Green chili	1014.21 ± 9.93	0.69 ± 0.18	6.34 ± 0.83	51.96 ± 7.81	113.70 ± 5.57	4.93 ± 3.84	5.82 ± 1.61	121.65 ± 0.07
6	XS n=20	Cucumber	523.17 ± 16.57	0.68 ± 0.23	4.24 ± 0.65	46.82 ± 3.56	22.65 ± 2.62	3.44 ± 1.68	5.77 ± 0.95	44.69 ± 0.16
		Tomato	510.26 ± 12.85	0.67 ± 0.32	3.65 ± 0.92	55.64 ± 3.49	31.85 ± 0.85	4.13 ± 2.28	5.32 ± 1.55	51.65 ± 0.10
		Bitter gourd	553.58 ± 15.86	0.69 ± 0.24	7.23 ± 0.84	42.17 ± 4.22	24.62 ± 3.12	3.65 ± 2.84	5.55 ± 2.96	43.56 ± 0.30

		Purple eggplant	592.97 ± 16.55	0.66 ± 0.25	6.19 ± 0.62	76.42 ± 2.95	52.32 ± 2.18	3.85 ± 2.20	5.22 ± 2.97	49.65 ± 0.32
		Green chili	553.05 ± 12.85	0.65 ± 0.26	5.22 ± 0.31	65.36 ± 2.55	49.61 ± 0.98	4.22 ± 1.50	5.31 ± 3.18	52.66 ± 0.15
		Tomato	798.32 ± 19.65	0.86 ± 0.23	6.15 ± 0.42	83.17 ± 2.95	46.12 ± 1.65	5.32 ± 0.98	6.41 ± 2.89	68.61 ± 0.16
		Green chili	816.49 ± 16.78	0.86 ± 0.30	7.26 ± 0.36	85.65 ± 2.54	88.95 ± 2.65	3.19 ± 1.01	7.64 ± 4.86	55.32 ± 0.11
7	YL n=20	Long purple eggplant	833.46 ± 15.94	0.83 ± 0.17	6.32 ± 0.41	67.61 ± 2.64	52.62 ± 0.84	4.98 ± 1.85	5.46 ± 2.65	49.65 ± 0.10
		Cucumber	735.14 ± 17.65	0.82 ± 0.25	5.16 ± 0.36	75.67 ± 3.48	49.62 ± 0.33	4.51 ± 1.64	4.68 ± 3.17	58.64 ± 0.18
		Cowpea	751.36 ± 16.68	0.80 ± 0.35	9.16 ± 0.39	85.61 ± 3.48	53.15 ± 2.85	3.65 ± 1.67	8.56 ± 4.17	59.64 ± 0.14
		Cucumber	961.35 ± 18.42	1.10 ± 0.24	8.12 ± 0.32	76.16 ± 2.55	43.36 ± 0.16	5.16 ± 1.49	5.68 ± 2.67	60.15 ± 0.18
		Chinese tarragon	1103.55 ± 16.16	2.26 ± 0.25	19.22 ± 0.25	95.62 ± 2.62	89.62 ± 0.64	4.96 ± 2.85	9.42 ± 1.06	159.63 ± 0.11
		Water spinach	1238.42 ± 11.52	2.33 ± 0.25	16.13 ± 0.37	103.45 ± 3.15	96.17 ± 0.54	3.59 ± 2.01	8.57 ± 0.94	177.65 ± 0.15
8	LS n=24	White tomato eggplant	1264.33 ± 14.62	1.30 ± 0.25	9.17 ± 0.22	83.19 ± 2.85	73.55 ± 0.95	4.68 ± 2.06	5.66 ± 1.55	120.64 ± 0.28
		Green chili	1164.68 ± 15.95	1.50 ± 0.33	8.65 ± 0.23	72.62 ± 3.54	73.15 ± 1.62	6.32 ± 2.06	7.61 ± 1.61	86.32 ± 0.36
		Tomato	1136.16 ± 12.85	1.31 ± 0.24	9.66 ± 0.31	69.32 ± 2.56	84.31 ± 2.06	5.16 ± 2.41	7.75 ± 1.19	81.55 ± 0.25
		Mini-cucumber	1439.16 ± 14.62	1.10 ± 0.29	8.65 ± 0.29	59.16 ± 3.98	69.42 ± 1.96	3.20 ± 1.55	6.63 ± 2.20	95.55 ± 0.26
		Cherry tomato	823.62 ± 15.65	1.35 ± 0.22	9.17 ± 0.61	88.61 ± 2.48	85.61 ± 0.58	6.52 ± 1.35	6.51±0.85	86.42 ± 0.26
9	GD n=20	Broccoli	995.31 ± 13.84	1.52 ± 0.24	7.61 ± 0.39	84.61 ± 2.68	87.16 ± 0.95	6.15 ± 1.55	7.24 ± 0.32	89.16 ± 0.22
		Purple ball eggplant	1003.55 ± 13.48	1.64 ± 0.35	5.61 ± 0.51	92.56 ± 3.62	92.38 ± 1.40	5.61 ± 1.85	7.12 ± 0.65	92.65 ± 0.24
		Green chili	1302.55 ± 15.65	1.11 ± 0.29	6.66 ± 0.27	68.48 ± 3.11	85.56 ± 2.94	6.31 ± 1.55	7.65 ± 1.52	83.05 ± 0.29

Table S9. Accumulation factor (AF) of target elements in vegetables of greenhouses in Jingmen City (mean ± SD) [1]

No.	Area	As	Cd	Cr	Cu	Hg	Ni	Pb	Zn	Total	Vegetable type
1	Zilingpu n=24	0.39 ± 0.43	0.47 ± 0.66	0.34 ± 0.57	0.24 ± 0.44	0.19 ± 0.48	0.17 ± 0.43	0.42 ± 0.08	0.50 ± 0.41	**2.72**	Green eggplant
		0.42 ± 1.13	0.71 ± 0.10	0.34 ± 0.09	0.21 ± 0.37	0.11 ± 0.11	0.15 ± 0.31	0.37 ± 1.32	0.40 ± 0.07	**2.71**	Yellow cucumber
		0.44 ± 0.92	0.19 ± 0.04	0.49 ± 0.22	0.28 ± 0.54	0.23 ± 0.20	0.14 ± 0.44	0.30 ± 0.49	0.61 ± 1.22	**2.68**	Cucumber
		0.42 ± 0.26	0.55 ± 0.72	0.10 ± 0.07	0.22 ± 0.96	0.23 ± 1.13	0.08 ± 0.34	0.37 ± 0.74	0.19 ± 0.03	**2.17**	Tomato
		0.33 ± 1.14	0.39 ± 0.56	0.14 ± 0.07	0.23 ± 0.97	0.07 ± 0.14	0.18 ± 0.45	0.38 ± 0.35	0.26 ± 0.10	**2.00**	Green chili
2	Zhanghe n=15	0.35 ± 0.19	0.47 ± 0.70	0.05 ± 0.06	0.22 ± 0.63	0.09 ± 0.13	0.10 ± 0.42	0.37 ± 0.30	0.29 ± 0.02	**1.94**	Small cushaw
		0.38 ± 0.18	0.31 ± 0.34	0.08 ± 0.28	0.30 ± 0.31	0.24 ± 1.24	0.17 ± 1.28	0.26 ± 0.43	0.13 ± 0.12	**1.86**	Purple long eggplant
		**1.06 ± 1.10**	0.30 ± 0.47	0.08 ± 0.10	0.22 ± 0.56	0.37 ± 1.38	0.21 ± 1.39	0.28 ± 0.24	0.10 ± 0.08	**2.62**	Green chili
		0.91 ± 0.25	0.23 ± 0.08	0.08 ± 0.42	0.17 ± 0.47	0.18 ± 0.26	0.14 ± 1.20	0.29 ± 0.41	0.48 ± 0.01	**2.48**	Cauliflower
4	Tuanlin n=24	0.43 ± 0.30	0.40 ± 2.78	**1.63 ± 0.23**	0.25 ± 0.28	0.11 ± 1.32	0.51 ± 0.56	0.36 ± 0.20	0.13 ± 0.02	**3.59**	Cherry tomato
		0.35 ± 0.11	0.24 ± 0.30	0.42 ± 0.47	0.19 ± 0.25	0.15 ± 0.09	0.12 ± 0.38	0.35 ± 0.46	0.18 ± 0.05	**2.00**	Long purple eggplant
		0.36 ± 0.19	0.45 ± 1.97	**2.79 ± 0.27**	0.22 ± 0.22	0.11 ± 0.15	0.39 ± 1.77	0.46 ± 2.31	0.21 ± 0.04	**2.49**	Pumpkin
		0.34 ± 1.11	0.38 ± 0.29	0.40 ± 0.31	0.26 ± 0.31	0.06 ± 0.28	0.12 ± 0.68	0.44 ± 0.64	0.18 ±0.01	**2.19**	Cucumber
		0.55 ± 0.15	0.29 ± 0.07	**1.68 ± 0.50**	0.34 ± 0.66	0.95 ± 0.78	0.26 ± 0.81	0.25 ± 5.76	0.66 ± 0.35	**3.22**	Lettuce
4	Zhongxiang n=20	**1.14 ± 0.09**	0.47 ± 0.31	0.49 ± 0.43	0.30 ± 0.36	0.82 ± 1.13	0.18 ± 0.67	0.23 ± 0.58	0.21 ± 0.07	**3.36**	Garland chrysanthemum
		0.61 ± 0.47	0.50 ± 0.55	0.08 ± 0.06	0.17 ± 0.21	0.23 ± 0.12	0.17 ± 0.35	0.37 ± 0.34	0.25 ± 0.03	**2.38**	Cauliflower
		**1.07 ± 0.48**	0.34 ± 0.20	0.11 ± 0.03	0.31 ± 0.41	0.58 ± 0.53	0.16 ± 0.32	0.38 ± 0.22	0.17 ± 0.01	**3.13**	Asparagus lettuce
		**1.24 ± 0.72**	**1.68 ± 0.60**	0.10 ± 0.32	0.23 ± 0.46	**1.48 ± 0.91**	0.19 ± 0.75	0.24 ± 0.06	0.60 ± 0.02	**4.70**	Chinese cabbage
		0.86 ± 0.51	0.55 ± 0.69	0.18 ± 0.11	0.46 ± 0.13	**1.65 ± 0.33**	0.17 ± 0.30	0.57 ± 0.54	0.27 ± 0.03	**3.26**	Green chili
5	Pengdun n=20	**1.47 ± 0.57**	0.84 ± 1.83	0.59 ± 0.16	0.54 ± 0.54	0.79 ± 6.81	0.22 ± 0.94	0.29 ± 0.21	0.93 ± 0.08	**3.75**	Cherry tomato
		0.42 ± 1.06	0.37 ± 0.11	0.07 ± 0.15	0.24 ± 0.27	0.23 ± 0.09	0.12 ± 0.84	0.27 ± 0.21	0.43 ± 0.02	**2.16**	Mini-cucumber
		0.37 ± 1.33	0.59 ± 1.09	0.07 ± 0.36	0.12 ± 0.34	0.25 ± 0.44	0.14 ± 1.25	0.26 ± 0.83	0.18 ± 0.10	**1.97**	Purple eggplant
		0.43 ± 0.77	0.33 ± 0.29	0.09 ± 0.13	0.27 ± 0.38	0.33 ± 0.23	0.13 ± 0.68	0.46 ± 0.71	0.33 ± 0.29	**2.38**	Green chili
6	Xinshi n=20	0.35 ± 0.75	0.50 ± 1.55	0.30 ± 0.12	0.17 ± 0.36	0.13 ± 0.62	0.13 ± 0.81	0.34 ± 0.37	0.39 ± 0.13	**2.31**	Cucumber
		0.36 ± 1.37	0.41 ± 0.85	0.34 ± 0.15	0.21 ± 0.56	0.15 ± 0.51	0.13 ± 0.46	0.36 ± 1.49	0.45 ± 0.08	**2.41**	Tomato
		0.39 ± 1.09	0.46 ± 0.95	0.32 ± 0.30	0.17 ± 0.56	0.12 ± 0.74	0.13 ± 0.79	0.40 ± 0.69	0.45 ± 0.06	**2.44**	Bitter gourd

		0.31 ± 4.63	0.49 ± 0.38	0.24 ± 0.11	0.23 ± 0.39	0.12 ± 0.50	0.14 ± 0.69	0.40 ± 0.68	0.40 ± 0.09	**2.31**	Purple eggplant
		0.32 ± 0.37	0.43 ± 0.82	0.38 ± 0.18	0.18 ± 0.50	0.15 ± 0.43	0.13 ± 0.72	0.37 ± 0.66	0.48 ± 0.05	**2.43**	Green chili
		0.32 ± 0.30	0.42 ± 0.95	0.30 ± 0.17	0.20 ± 0.49	0.21 ± 0.47	0.18 ± 0.94	0.34 ± 0.36	0.56 ± 0.05	**2.52**	Tomato
		0.33 ± 0.74	0.43 ± 0.52	0.38 ± 0.11	0.23 ± 0.43	0.24 ± 1.00	0.16 ± 0.96	0.33 ± 1.11	0.47 ± 0.05	**2.58**	Green chili
7	Yonglong n=20	0.32 ± 0.72	0.44 ± 0.45	0.33 ± 0.09	0.23 ± 0.49	0.23 ± 0.90	0.17 ± 0.61	0.36 ± 0.82	0.45 ± 0.07	**2.52**	Long purple eggplant
		0.30 ± 0.43	0.39 ± 1.04	0.34 ± 0.08	0.24 ± 0.29	0.26 ± 0.80	0.17 ± 0.53	0.31 ± 0.33	0.47 ± 0.06	**2.48**	Cucumber
		0.37 ± 0.50	0.44 ± 0.55	0.33 ± 0.10	0.25 ± 0.43	0.29 ± 0.59	0.18 ± 0.55	0.34 ± 0.57	0.55 ± 0.07	**2.74**	Cowpea
		0.40 ± 0.46	0.42 ± 0.33	0.22 ± 0.09	0.33 ± 0.40	0.32 ± 0.74	0.18 ± 0.54	0.30 ± 0.39	0.47 ± 0.07	**2.63**	Cucumber
		0.39 ± 0.41	0.29 ± 0.39	0.25 ± 0.09	0.35 ± 0.66	0.33 ± 0.79	0.19 ± 0.85	0.36 ± 0.28	0.38 ± 0.06	**2.52**	Chinese tarragon
		0.34 ± 0.99	0.30 ± 0.33	0.23 ± 0.10	0.34 ± 0.34	0.31 ± 0.73	0.16 ± 0.80	0.44 ± 0.56	0.39 ± 0.11	**2.50**	Water spinach
8	Lishi n=24	0.39 ± 0.39	0.32 ± 0.54	0.23 ± 0.08	0.33 ± 0.31	0.29 ± 0.70	0.18 ± 0.54	0.34 ± 0.36	0.46 ± 0.02	**2.52**	White tomato eggplant
		0.35 ± 0.32	0.33 ± 0.34	0.21 ± 0.11	0.35 ± 0.47	0.31 ± 0.51	0.19 ± 1.14	0.37 ± 0.35	0.44 ± 0.04	**2.54**	Green chili
		0.44 ± 0.54	0.38 ± 0.35	0.20 ± 0.08	0.35 ± 0.55	0.30 ± 0.24	0.18 ± 0.52	0.36 ± 0.31	0.55 ± 0.04	**2.76**	Tomato
		0.39 ± 0.74	0.41 ± 0.36	0.16 ± 0.11	0.36 ± 0.25	0.35 ± 0.41	0.22 ± 1.04	0.32 ± 0.41	0.50 ± 0.07	**2.71**	Mini-cucumber
		0.34 ± 0.88	0.39 ± 0.40	0.16 ± 0.12	0.35 ± 0.41	0.39 ± 0.27	0.23 ± 0.81	0.32 ± 0.41	0.38 ± 0.06	**2.57**	Cherry tomato
9	Guandang n=20	0.34 ± 0.49	0.45 ± 0.41	0.17 ± 0.09	0.35 ± 0.43	0.32 ± 0.30	0.21 ± 0.69	0.29 ± 0.49	0.32 ± 0.04	**2.43**	Broccoli
		0.38 ± 0.29	0.41 ± 0.34	0.18 ± 0.14	0.35 ± 0.39	0.36 ± 0.23	0.21 ± 0.47	0.30 ± 0.45	0.32 ± 0.05	**2.50**	Purple ball eggplant
		0.41 ± 0.26	0.36 ± 0.39	0.18 ± 0.14	0.32 ± 0.42	0.35 ± 0.21	0.20 ± 0.53	0.26 ± 0.38	0.29 ± 0.06	**2.38**	Green chili
Average value		0.48 ± 0.68	0.44 ± 0.61	0.28 ± 0.18	0.27 ± 0.44	0.25 ± 0.68	0.17 ± 0.72	0.34 ± 0.65	0.37 ± 0.10	**2.60**	

[1] AF values higher than 1.00 have been listed in bold.

Table S10. Principal component analysis (PCA) of eight target elements in different samples collected from nine study areas in Jingmen City.

	**Element**	**Principal component**		
		1	2	3
Soil	As	0.25892	0.51598	0.23596
	Cd	0.37397	0.27154	-0.03722
	Cr	0.39673	-0.25026	-0.38999
	Cu	0.52814	-0.20011	0.16248
	Hg	-0.03631	0.43695	0.3918
	Ni	0.18414	0.53824	-0.3389
	Pb	0.50188	-0.08782	-0.16422
	Zn	0.26395	-0.26272	0.68492
Vegetable edible part	As	0.2052	0.59594	-0.00968
	Cd	0.21175	0.56298	-0.00156
	Cr	0.26322	-0.36646	-0.50727
	Cu	0.46505	-0.16211	0.28457
	Hg	0.49106	0.16064	0.02237
	Ni	0.47299	-0.29284	-0.16962
	Pb	0.18342	-0.22047	0.77177
	Zn	0.35915	0.08468	-0.1916
Vegetable leaf	As	0.32491	-0.13074	0.45428
	Cd	0.36689	-0.33286	0.26633
	Cr	0.34385	-0.19894	0.4138
	Cu	0.35684	-0.41657	-0.41349
	Hg	0.43667	0.01057	-0.53342
	Ni	0.20959	0.66247	0.20242
	Pb	0.41067	0.26735	0.06266
	Zn	0.33316	0.38543	-0.22596

Table S11. Characteristic value and accumulative contribution of PCA.

	Principal component	Eigenvalue	Contribution rate of variance	Total contribution rate of variance
Soil	1	2.81283	35.16 %	35.16 %
	2	1.95003	24.38 %	59.54 %
	3	1.30566	16.32 %	75.86 %
	4	0.71227	8.90 %	84.76 %
	5	0.58457	7.31 %	92.07 %
	6	0.32878	4.11 %	96.18 %
	7	0.21708	2.71 %	98.89 %
	8	0.08879	1.11 %	100.00 %
Vegetable edible part	1	3.06137	38.27 %	38.27 %
	2	1.50283	18.79 %	57.05 %
	3	1.15892	14.49 %	71.54 %
	4	0.8805	11.01 %	82.55 %
	5	0.62159	7.77 %	90.31 %
	6	0.3461	4.33 %	94.64 %
	7	0.23902	2.99 %	97.63 %
	8	0.18968	2.37 %	100.00 %
Vegetable leaf	1	2.83955	35.49 %	35.49 %
	2	1.45891	18.24 %	53.73 %
	3	1.06679	13.33 %	67.07 %
	4	0.80339	10.04 %	77.11 %
	5	0.63664	7.96 %	85.07 %
	6	0.5277	6.60 %	91.66 %
	7	0.40507	5.06 %	96.73 %
	8	0.26196	3.27 %	100.00 %
